# The Founder Strains of the Collaborative Cross Express a Complex Combination of Advantageous and Deleterious Traits for Male Reproduction

**DOI:** 10.1534/g3.115.020172

**Published:** 2015-10-13

**Authors:** Fanny Odet, Wenqi Pan, Timothy A. Bell, Summer G. Goodson, Alicia M. Stevans, Zianing Yun, David L. Aylor, Chia-Yu Kao, Leonard McMillan, Fernando Pardo-Manuel de Villena, Deborah A. O’Brien

**Affiliations:** * Department of Cell Biology and Physiology, University of North Carolina, Chapel Hill, North Carolina 27599; † Department of Genetics, University of North Carolina, Chapel Hill, North Carolina 27599; ‡ Lineberger Comprehensive Cancer Center, University of North Carolina, Chapel Hill, North Carolina 27599; § Carolina Center for Genome Sciences, University of North Carolina, Chapel Hill, North Carolina 27599; ** Department of Biological Sciences, North Carolina State University, Raleigh, North Carolina 27695; †† Department of Computer Science, University of North Carolina, Chapel Hill, North Carolina 27599

**Keywords:** sperm, motility, vacuole, Collaborative Cross

## Abstract

Surveys of inbred strains of mice are standard approaches to determine the heritability and range of phenotypic variation for biomedical traits. In addition, they may lead to the identification of novel phenotypes and models of human disease. Surprisingly, male reproductive phenotypes are among the least-represented traits in the Mouse Phenome Database. Here we report the results of a broad survey of the eight founder inbred strains of both the Collaborative Cross (CC) and the Diversity Outbred populations, two new mouse resources that are being used as platforms for systems genetics and sources of mouse models of human diseases. Our survey includes representatives of the three main subspecies of the house mice and a mix of classical and wild-derived inbred strains. In addition to standard staples of male reproductive phenotyping such as reproductive organ weights, sperm counts, and sperm morphology, our survey includes sperm motility and the first detailed survey of testis histology. As expected for such a broad survey, heritability varies widely among traits. We conclude that although all eight inbred strains are fertile, most display a mix of advantageous and deleterious male reproductive traits. The CAST/EiJ strain is an outlier, with an unusual combination of deleterious male reproductive traits including low sperm counts, high levels of morphologically abnormal sperm, and poor motility. In contrast, sperm from the PWK/PhJ and WSB/EiJ strains had the greatest percentages of normal morphology and vigorous motility. Finally, we report an abnormal testis phenotype that is highly heritable and restricted to the WSB/EiJ strain. This phenotype is characterized by the presence of a large, but variable, number of vacuoles in at least 10% of the seminiferous tubules. The onset of the phenotype between 2 and 3 wk of age is temporally correlated with the formation of the blood-testis barrier. We speculate that this phenotype may play a role in high rates of extinction in the CC project and in the phenotypes associated with speciation in genetic crosses that use the WSB/EiJ strain as representative of the *Mus muculus domesticus* subspecies.

The ability to reproduce is a defining characteristic of living organisms. In mammals and many other species, reproductive success depends on both genetics and environmental factors and their interactions. Genetic factors in each parent and interactions between factors in both parents contribute to reproductive success. Despite the importance of genetics in reproductive phenotypes, there is a scarcity of suitable models for mapping loci associated with fertility-related traits.

The clinical diagnosis of infertility in men centers on standardized semen analysis, including assessment of sperm concentration, morphology, and motility (World Health Organization 2010). Although further tools are sometimes used to evaluate known causes of reduced sperm quality (such as endocrine disorders or sex chromosome abnormalities), assisted reproductive techniques often are used to achieve fertilization without identifying the cause of the defect. The list of gene defects that are associated with male infertility is growing and includes several recognized syndromes with systemic effects, such as cystic fibrosis (Dohle *et al.* 2005; Hotaling and Carrell 2014). Nevertheless, 30–50% of male infertility is considered idiopathic (Lipshultz and Lamb 2007).

The mouse provides a powerful experimental model to address some of the limitations of human studies and has been used successfully in hundreds of studies to dissect the genetic components of biomedical traits (Mott and Flint 2013; Peters *et al.* 2007). Targeted gene disruptions in mice have led to the rapid expansion of the list of candidate gene mutations and polymorphisms associated with infertility (Matzuk and Lamb 2008). In addition, large-scale mutagenesis programs have used *N*-ethyl-*N*-nitrosourea to create random mutations in mice that affect reproduction (Handel *et al.* 2006; Kennedy and O’Bryan 2006). Both approaches have identified many more mutations that selectively affect fertility in males than in females. This difference may be related to the large number of genes that are selectively expressed during spermatogenesis (Schultz *et al.* 2003; Wu *et al.* 2004; Chalmel *et al.* 2007).

A key resource for mouse research is the existence of hundreds of inbred strains derived from multiple sources and with a wide variety of genetic makeups (Didion and Pardo-Manuel de Villena 2013). Because individuals from any given inbred strain can be replicated at will, it is possible to use these strains to precisely characterize as many phenotypes as desired, to determine the relationships between them, and to disentangle the contribution of both male and female factors to overall reproductive success. Inbred strains, or their derivatives, can then be used to set up experimental crosses to optimize the chance of identifying quantitative trait loci (QTL) and eventually genes associated with the traits of interest. Ideally, every possible mouse inbred strain would be phenotyped to facilitate the development of new mouse models for human disease and to select the optimal combination of parents in experimental crosses. In practice, only a small subset can be phenotyped due to physical and budgetary constraints. Therefore, it is important to carefully select inbred strains so that the work has the greatest impact in both model development and genetic mapping.

Phenotypic characterization of inbred strains that are used to generate genetic reference populations (GRP) is especially attractive because genetic mapping in GRPs can be performed at relatively high precision in the absence of additional genotyping. Because individuals from GRPs are also inbred, it is possible to integrate multiple phenotypes (Threadgill *et al.* 2002). The most commonly used GRPs are recombinant inbred (RI) lines. Traditional RI lines are produced by crossing two inbred strains to produce F2 mice, followed by sibling matings for many generations to establish new isogenic strains (Peters *et al.* 2007). Although RI lines have been widely used for genetic mapping, they are typically derived from a two classical inbred strains, and these classical inbred strains have a large fraction of their genomes that are identical by descent (Yang *et al.* 2011), which limits the genetic diversity of these lines. To overcome these limitations, the Collaborative Cross (CC) Consortium has generated a panel of multiparental RI lines derived from five classical inbred strains (A/J, C57BL/6J, 129S1/SvImJ, NOD/ShiLtJ, and NZO/HILtJ) and three wild-derived strains (CAST/EiJ, PWK/PhJ, and WSB/EiJ) (Collaborative Cross Consortium 2012). These eight founder strains were selected based on availability, known genetic diversity (in 2004), phenotypic diversity, and breeding performance. Subsequently, high-density genotyping and whole-genome sequence has been generated and these data are publicly available (Yang *et al.* 2011; Keane *et al.* 2011). The addition of the wild-derived strains enabled the CC to capture 90% of genetic variation present in laboratory stocks of *Mus musculus* (Roberts *et al.* 2007), with the added advantage of making the spatial distribution of genetic variation quasi-uniform (Aylor *et al.* 2011; Collaborative Cross Consortium 2012). Finally, the same eight founders were used to generate a sister resource known as the Diversity Outbred population (Svenson *et al.* 2012).

During the generation of the CC mice, a large fraction of the lines ceased to produce offspring leading to an extremely high extinction rate (>80%; F. Pardo-Manuel de Villena, unpublished data). Breeding performance decreased dramatically after the start of inbreeding but stabilized after generation G2:F7 (Collaborative Cross Consortium 2012). Hybrid sterility and reproductive incompatibility between pairs of CC founder strains are known to occur (Chesler *et al.* 2008). However, most the extinction is expected to be due to fixation of incompatible alleles at different loci (Collaborative Cross Consortium 2012). Importantly, a large portion of the extinction is due to male infertility (F. Pardo-Manuel de Villena and D. O'Brien, unpublished data).

To better understand the genetics of male reproductive traits in the CC population, it is important to characterize the eight founder inbred strains of the CC for these traits. Here we report body and reproductive organ weights (testis, epididymis + vas deferens, seminal vesicles), sperm counts, sperm morphology, sperm motility, sperm lactate production, and testis histology from 248 adult males from all eight founders of the CC. We estimate a wide range of heritability values among these traits. In addition, we report reproductive phenotypes unique to specific CC founders. These data provide the most comprehensive picture of male reproductive phenotypes of the eight founder strains of the CC to date. This study should facilitate a better understanding of varied breeding performance in the CC, as well as interpretation of genetic mapping of male reproductive traits in the population.

## Materials and Methods

### Mice

Reproductive phenotypes were determined for adult males (≥10 wk of age) from the eight founder strains (A/J, C57BL/6J, 129S1/SvImJ, NOD/ShiLtJ, NZO/H1LtJ, CAST/EiJ, PWK/PhJ and WSB/EiJ) used to generate the multiparental CC panel of RI lines. All animals were bred at the University of North Carolina from parents that were fewer than six generations removed from founders obtained from the Jackson Laboratory. Mice were housed in standard 20 × 30-cm ventilated polysulfone cages with laboratory grade Bed-O-Cob bedding. Water and Purina Prolab RMH3000 were available *ad libitum*, and a small section of PVC pipe was present in each cage for enrichment. Multiple phenotypes were determined for each animal (Supporting Information, Table S1 and Table S2). In total we phenotyped 248 mice with a range of ages (Figure S1). At least nine mice from each strain were examined for each phenotype, except sperm lactate production (≥4 mice/strain). Testis histology was also assessed in additional juvenile C57BL/6J and WSB/EiJ mice (14−42 d old). Forty-seven F1 hybrid mice between the WSB/EiJ strain and the other seven founders [two (A/JxWSB/EiJ)F1, four (WSB/EiJxA/J)F1, two (C57/BL6JxWSB/EiJ)F1, four (WSB/EiJxC57BL/6J)F1, four (129S1/SvImJxWSB/EiJ)F1, four (WSB/EiJx129S1/SvImJ)F1, four (NOD/ShiLtxWSB/EiJ)F1, three (WSB/EiJxNOD/ShiLtJ)F1, two (NZO/HILtJxWSB/EiJ)F1, five (CAST/EiJxWSB/EiJ)F1, five (WSB/EiJxCAST/EiJ)F1, three (PWK/PhxWSB/EiJ)F1, and five (WSB/EiJxPWK/PhJ)F1] were generated at the University of North Carolina and phenotyped for the vacuole phenotype. All procedures involving animals were performed according to the Guide for the Care and Use of Laboratory Animals with prior approval by the Institutional Animal Care and Use Committee within the AAALAC accredited program at the University of North Carolina at Chapel Hill (Animal Welfare Assurance Number: A-3410-01).

Forty-five mice have been reported previously as part of a study on the side effects of antipsychotic drugs haloperidol and clozapine (Crowley *et al.* 2012). Nine mice were treated with clozapine (one A/J, four C57BL/6J, one 129S1/SvImJ, one NOD/ShiLtJ, and two WSB/EiJ), 12 mice were treated with haloperidol (one A/J, three C57BL/6J, two 129S1/SvImJ, two NOD/ShiLtJ, one NZO/H1LtJ, one PWK/PhJ, and two WSB/EiJ), and 24 mice were treated with vehicle and used as controls (two A/J, eight C57BL/6J, four 129S1/SvImJ, three NOD/ShiLtJ, one NZO/H1LtJ, one CAST/EiJ, one PWK/PhJ, and four WSB/EiJ). Using analysis of variance (ANOVA) and exact *t*-tests, we did not observe any significant drug effect for any of the reproductive phenotypes reported here either globally or within any of the strains.

### Reproductive organ weights

**Figure 1:**
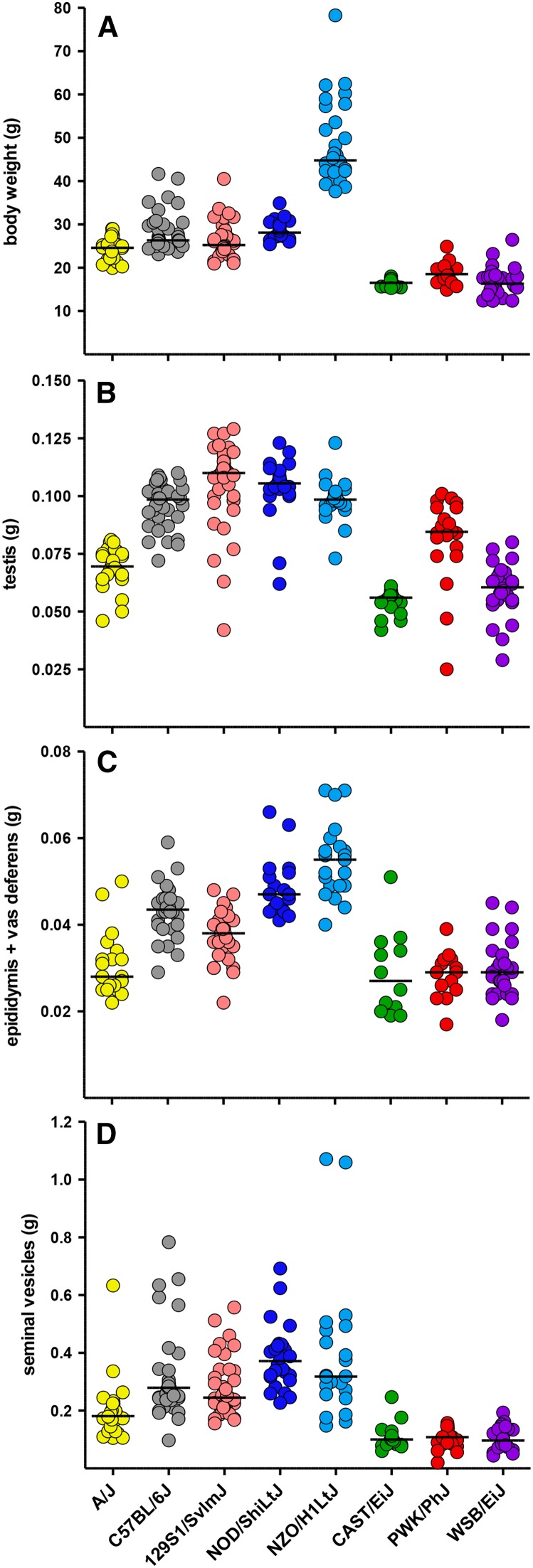
Body and reproductive organ weights in males from the eight founder strains of the CC. The colors represent the eight founder strains as follows: A/J, yellow; C57BL/6J, gray; 129S1/SvImJ, pink; NOD/ShiLtJ, dark blue; NZO/H1LtJ, light blue; CAST/EiJ, green; PWK/PhJ, red; and WSB/EiJ, purple. Each male is represented by a circle. All weights are in grams, including (A) body weight, (B) mean testis weight, (C) mean weight of the epididymis with attached vas deferens, and (D) weight of the seminal vesicles. The same order and color scheme are used throughout this article. The black horizontal lines in all graphs represent the median for each strain.

Each male was killed by CO_2_ asphyxiation followed by cervical dislocation and the carcass was weighed. After careful dissection, wet weights were recorded for each testis and each epididymis with attached vas deferens. Mean weights for these organs are shown in Figure 1. Seminal vesicle weights also were recorded as an indication of endocrine status.

### Testis histology

After fixation in Bouins solution, one testis/mouse was cut in half horizontally and embedded in paraffin. Testis sections (8 µm) were stained with periodic acid-Schiff reagent and counterstained with hematoxylin to facilitate analysis and staging of spermatids based on acrosomal morphology (Russell *et al.* 1990). Composite images showing a complete transverse section from each testis (20× magnification) were generated using an Olympus BX51 microscope equipped with an Olympus DP72 digital camera, a motorized stage and MetaMorph automation & image analysis software (Molecular Devices, Sunnyvale, CA). MetaMorph measurements of the height, width, perimeter, and area of each section also were recorded. Each composite image was examined in detail to determine the number of seminiferous tubule cross sections and the number of tubules exhibiting defects, including vacuoles, abnormal or degenerating germ cells, immature germ cells in the lumen, and significant germ cell loss.

### Imaging tools

The digitized composite testis images were annotated with a custom interactive image analysis package that we developed. The first phase of the analysis was the automatic off-line identification of seminiferous tubule centers based on a random-forest classifier that used local-color histograms at each pixel as features. The model was trained with a small subset (<0.1%) of hand-annotated examples and was able to accurately localize more than 95% of all rete testis and tubule cross sections with an acceptable type 1 error rate (fewer than 2% per image). Once tubule centers were automatically found, we refined the results using a Web-based interactive tool to correct both missed and mislabeled centers. In addition, we used the interactive tool to annotate features of each tubule, including abnormalities such as vacuoles and the loss of germ cells at successive stages of differentiation. All of the final annotation information (tubule centers and classes) was kept in a database and manually verified in separate and multiple reviews. Tubule radii are automatically estimated as one half of the distance to the nearest neighboring tubule. Figure S2 provides a representative example of these tools. All images are available at the Systems Genetics Web site at UNC (http://database.csbio.unc.edu/Infertility) that hosts information on the CC. We will provide access to all of the histology images with the labels and annotations as described in the manuscript. This includes tubule counts, tubule-type labels, and minor-axis radii. Instructions for accessing all of the primary data presented will also be available at that Web site.

### Sperm counts, motility, and morphology

The right cauda epididymis was clipped with iris scissors in 500 μL of phosphate-buffered saline and incubated for 10 min in a 37° incubator. Sperm were extruded from the cauda with fine forceps. The sperm suspension was transferred to a microfuge tube and the collection well was rinsed with an additional 500 μL of phosphate-buffered saline. After appropriate dilutions, sperm were counted using a hemocytometer.

The left cauda epididymis was clipped with iris scissors and transferred to a 37° incubator (5% CO_2_ in air), allowing sperm to swim out for 10 min into 1 mL of human tubule fluid medium + 5 mg/mL bovine serum albumin (HTF; Goodson *et al.* 2011). This medium is based on the composition of human oviductal fluid (Quinn *et al.* 1985) and supports *in vitro* fertilization in a variety of mouse strains (Byers *et al.* 2006). Sperm suspensions were diluted with HTF and incubated for 2 hr at 37° under 5% CO_2_ in air. Motility was assessed at 30-min intervals throughout this *in vitro* capacitation period by computer-assisted sperm analysis (CASA) via a Hamilton Thorne CEROS imaging system with version 12.3H IVOS software (Goodson *et al.* 2011). Leja chambers (100 µm-depth; Leja Products BV, Nieuw-Vennep, The Netherlands) were used for these analyses. Sperm tracks (90 frames, 1.5 sec) and kinetic parameters for individual sperm were captured at 60 Hz using motility analysis parameters (mouse 2) recommended by Hamilton Thorne Biosciences, except that slow sperm were considered motile. Tracks in 10 fields were typically recorded for each mouse, along with mean values for average path velocity (VAP, µm/sec), straight line velocity (VSL, µm/sec), curvilinear velocity (VCL, µm/sec), amplitude of lateral head displacement (ALH, µm), beat cross frequency (BCF, Hz), straightness (STR), and linearity (LIN). Sperm from multiple strains were typically assessed in each experiment.

CASAnova, a support vector machines program based on CASA parameters of sperm from CD1 mice (Goodson *et al.* 2011), was used to classify individual sperm into one of five motility groups: progressive, intermediate, hyperactivated, slow, and weakly motile. For each inbred strain, both the number and percentage of motile sperm in these five categories was determined at each of the 30-min motility time points throughout the 2-hr capacitation period.

To assess morphology, 10-μL aliquots of the HTF sperm suspension were spread onto positively charged slides and allowed to air dry briefly until moisture had just evaporated. These samples were then fixed with −20° methanol for 10 min, air dried, and stored at −20°. Acrosomes were stained with peanut agglutinin conjugated to a fluorescent tag (Alexa Fluor 488; Invitrogen) (Lee *et al.* 2008) before microscopic analysis. By the use of phase contrast optics, sperm (>100/sample) were scored as having normal morphology, abnormal head shape, abnormal tail bending (≥90°), or broken tails (severed at the head/neck junction or at more distal locations along the length of the flagellum). Fluorescent, phase contrast, and differential interference contrast images were archived for each sample.

### Sperm lactate production

Sperm lactate production was measured with a spectrophotometric assay that monitors the accumulation of NADH during the oxidation of lactate to pyruvate by lactate dehydrogenase (Pesce *et al.* 1975; Danshina *et al.* 2001). Sperm were incubated for 2 hr using our standard conditions for capacitation, except that lactate and pyruvate were omitted from the HTF medium. Duplicate aliquots were removed at 0 and 2 hr to measure lactate accumulation in the medium. After sperm was removed by centrifugation, samples were added to the assay buffer (pH 9.0) containing 1 mM NAD^+^ and 10 U/mL lactate dehydrogenase from rabbit muscle (Sigma-Aldrich, St. Louis, MO) and incubated for 2 hr at 25°. The concentration of lactate in the sample was proportional to the increase in absorbance at 340 nm, as NAD^+^ was reduced to NADH. Sperm in each sample were counted and lactate production (μmole/10^8^ sperm) during the 2-hr incubation period was calculated.

### Statistical analysis

We used modified *z* scores to identify outlier strains for each trait measured using the equation:

z=0.6745×(sample median−population median)/(median absolute deviation in each strain)

If the median absolute deviation equaled zero we used the equation:

z=0.98×(sample median−population median)/(mean absolute deviation in each strain)

Modified *z* scores larger than 1 and 3 were classified as high and very high, respectively. *z* scores lower than −1 and −3, were classified as low and very low, respectively (Table S3). The modified *z* score is more robust than the standard *z* score because it uses the median instead of the mean and thus is less influenced by outliers within each strain. Pearson correlations between all phenotypes were calculated in R (R Core Team; http://www.R-project.org). Missing pairwise values were disregarded. We used one-way ANOVA to test whether age had an effect on any of the phenotypes. Additionally, we fit a two-way ANOVA including the effects of strain, age, and interaction term.

Heritability is defined as the proportion of the overall variability observed in that trait that is due to inherited genetic factors. The “broad-sense heritability” (*h^2^*) was estimated using the equation

$$h^{2}=V_{A}/\left(V_{A}+V_{E}\right)$$

where the between-strains variance (VA) is divided by the total variance. The total phenotypic variance is the sum of VA and the within-strain variance (VE), which reflects the effects of all other biological and experimental factors (Falconer and Mackay 1996).

### Data availability

All inbred strains are available from the Jackson Laboratory. Table S1 contains primary data for all 248 mice that were phenotyped. Table S2 provides sperm motility data throughout a 2-hr time course for 116 of these mice. Table S3 provides the means, medians and z scores for traits in Table 1. Table S4 contains the correlations between traits shown in Table 1. Table S5 provides the p values for two-way ANOVA with age, strain and interaction between both. Testis histology images and annotations for all samples can be found at http://database.csbio.unc.edu/Infertility.

**Table 1:** Phenotypes measured in the eight founder strains of the Collaborative Cross

Trait	No. Samples	Range of Samples per Founder	Figure	*h^2^*
Age	248	na	Figure S1	na
Weights				
Body	231	19−38	Figure 1A	0.84
Mean testis	218	20−38	Figure 1B	0.71
Mean epididymis + vas deferens	170	12−30	Figure 1C	0.70
Seminal vesicles	217	20−38	Figure 1D	0.51
Histology				
Number of tubules	208	17−36	Figure 3A	0.50
Mean tubule radius	208	17−36	Figure 3B	0.57
Seminiferous epithelium length	208	17−36	Figure 3C	0.48
# of tubules with vacuoles	207	16−36	Figure 4A	0.35
# of tubules with many vacuoles	207	16−36	Figure 4B	0.39
# of tubules with germ cell loss	141	12−26	Figure S4	0.15
# of tubules with abnormal germ cells	207	16−36	Figure S4	0.12
# of tubules with germ cell sloughing	207	16−36	na	0.11
Sperm count				
10^6^/mouse	171	9−32	Figure 6A	0.38
10^3^/mg testis	159	9−30	Figure 6B	0.27
10^3^/seminiferous epithelium length	152	8−28	Figure 6C	0.22
Sperm morphology				
% normal	111	10−19	Figure 7A	0.47
% abnormal head shape	111	10−19	Figure 7B	0.42
% abnormal tail bending	111	10−19	Figure 7C	0.34
% broken tails	111	10−19	Figure 7D	0.07
Sperm motility t = 10 min				
% motile	116	10−18	Figure 8A	0.42
VCL	116	10−18	Figure 8C	0.68
% vigorous	116	10−18	Figure 9	0.53
% hyperactive	116	11−18	Figure 9	0.33
Sperm motility t = 90 min				
% motile	105	10−16	Figure 8B	0.60
VCL	105	10−16	Figure 8D	0.71
% vigorous	105	10−16	Figure 9	0.73
% hyperactive	105	10−16	Figure 9	0.40
Sperm metabolism				
Lactate production	61	4−11	Figure 10	0.29

For each trait, the table lists the total number of mice sampled, the range of samples measured/founder strain, the figure reporting values for that trait and its narrow-sense heritability (*h^2^*) as defined in the *Materials and Methods*. na, not available; VCL, curvilinear velocity.

## Results

To establish a comprehensive framework for the genetic analysis of male reproduction and fertility in the CC, we assessed reproductive organ weights, testis histology, sperm counts, and multiple characteristics of sperm quality in adult males from each of the eight founder strains. Table 1 lists the phenotypes collected in this study and their heritability (*h^2^*). The primary data for each mouse is provided in Table S1 and Table S2. Mice (>20/strain) examined in this study were less than 16 mo old (range 70−461 days of age). Similar age ranges were included for each strain (Figure S1), and both mean and median ages were not statistically different among strains. Table S3 provides mean, median, and SD, as well as the modified *z* score for each trait. To simplify inspection of this table, significant modified *z* scores (see the section *Materials and Methods*) are color coded to denote whether the direction of the scores is expected to have a advantageous (light, standard, and dark blue) or deleterious (yellow, orange, and red) effect on male reproduction. In the following sections, outlier strains for each phenotype are identified by the *z* score, whereas comparisons between or across selected strains use the test statistics described in the corresponding section. Finally, Table S4 summarizes the correlation between all phenotypes collected in this study, and Table S5 provides the significance of a two-way ANOVA with age, strains and their interaction.

### Correlation of reproductive organ weights with body weight and age

As expected, body weight varied significantly between strains (Figure 1A) and was highly heritable (Table 1). The three wild-derived strains, CAST/EiJ, PWK/PhJ, and WSB/EiJ, had significantly lower body weights than all classical inbred strains (Table S3). Both NZO/H1LtJ and NOD/ShiLtJ males were significantly heavier than other strains, whereas the other three classical inbred strains (A/J, C57BL/6J, and 129S1/SvImJ) had intermediate body weights. Body weight was positively correlated with age (Table S4), and there is a significant interaction between age and strain (Table S5).

Reproductive organ weights, including testis (Figure 1B), epididymis plus vas deferens (E+V, Figure 1C), and seminal vesicles (SV, Figure 1D), had high heritability (0.71, 0.70, and 0.51, respectively; Table 1) and were positively correlated with body weight (Table S4). The wild-derived strains had significantly lower reproductive organ weights, except for testis weight in PWK/PhJ mice (Table S3). A/J mice had the smallest reproductive organ weights among the classical inbred strains. Testis weights were significantly greater in NOD/ShiLtJ, NZO/H1LtJ, and 129S1/SvImJ males, and three of the classical inbred strains (NOD/ShiLtJ, NZO/H1LtJ, and C57BL/6J) had significantly greater weights for E+V and/or SV. SV weights were positively correlated with age and there is age by strain interaction (Table S5). Testis weights were not correlated with age, and E+V weights showed only a marginal correlation (Table S5).

### Testis histology

To facilitate detailed histological analyses, we constructed composite testis images of complete transverse sections stained with periodic acid-Schiff reagent and hematoxylin for more than 200 mice. Imaging tools were developed to automatically identify and count the seminiferous tubule cross sections in each composite image, estimate the mean radius length, scan individual tubules at greater magnification, and enable manual labeling of tubules as normal or exhibiting common abnormalities (Figure S2). These tools facilitated quantitative assessment of heritability and variation for the number and radius of seminiferous tubule cross sections and the number of tubules with defects including the presence of vacuoles, germ cell loss, sloughing of earlier stage germ cells into the tubule lumen, and the presence of abnormal germ cells.

**Figure 2:**
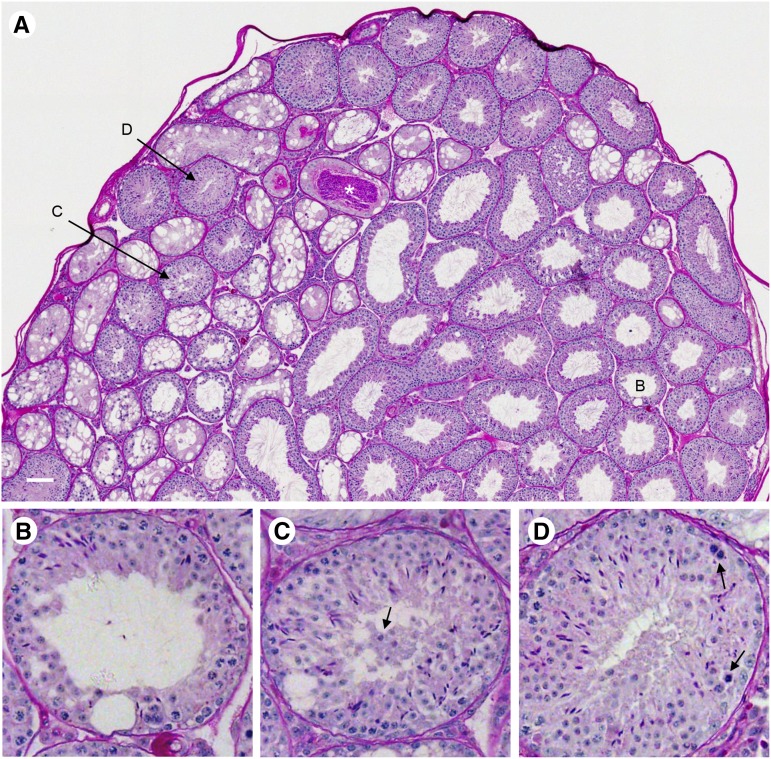
Testis histology. (A) A composite testis image for a WSB/EiJ male (HH0039 in Table S1) shows numerous seminiferous tubules with extensive vacuolization in the left half of the section. These tubules typically have very few spermatogenic cells. The white asterisk marks a Sertoli cell-only tubule with a large number of sperm in the lumen. Tubules B, C, and D are shown at greater magnification below (A). The white bar in lower left corner = 100 μm. (B) This tubule has a single large vacuole in the lower half of the seminiferous epithelium, with only a small number of elongated spermatids remaining is the upper left. (C) Isolated vacuoles are also apparent in this tubule, and earlier-stage germ cells are present in the tubule lumen. (D) Abnormal germ cells with very condensed nuclei and darker cytoplasm (arrows) are present in this tubule. This category also includes multinucleated germ cells (not shown).

Figure 2A shows approximately half of one section from a WSB/EiJ testis, illustrating representative examples of the defects we observed. Although most seminiferous tubule cross sections in this image appear normal, several tubules exhibit extensive vacuolization and germ cell loss. One vacuolated tubule has an unusually large accumulation of sperm in the lumen (white asterisk). Isolated vacuoles are apparent in other tubules, including those labeled as B and C. At greater magnification, tubule B also shows germ cell loss, particularly of late-stage elongated spermatids (Figure 2B). An abnormal blood vessel, with an asymmetric diameter and accumulation of periodic acid-Schiff-stained material, is seen below tubule B. Sloughing of earlier stage germ cells into the lumen was noted in tubule C (Figure 2C). Abnormal germ cells, typically degenerating or multinucleated, were occasionally observed in this section (tubule D, shown at greater magnification in Figure 2D).

**Figure 3:**
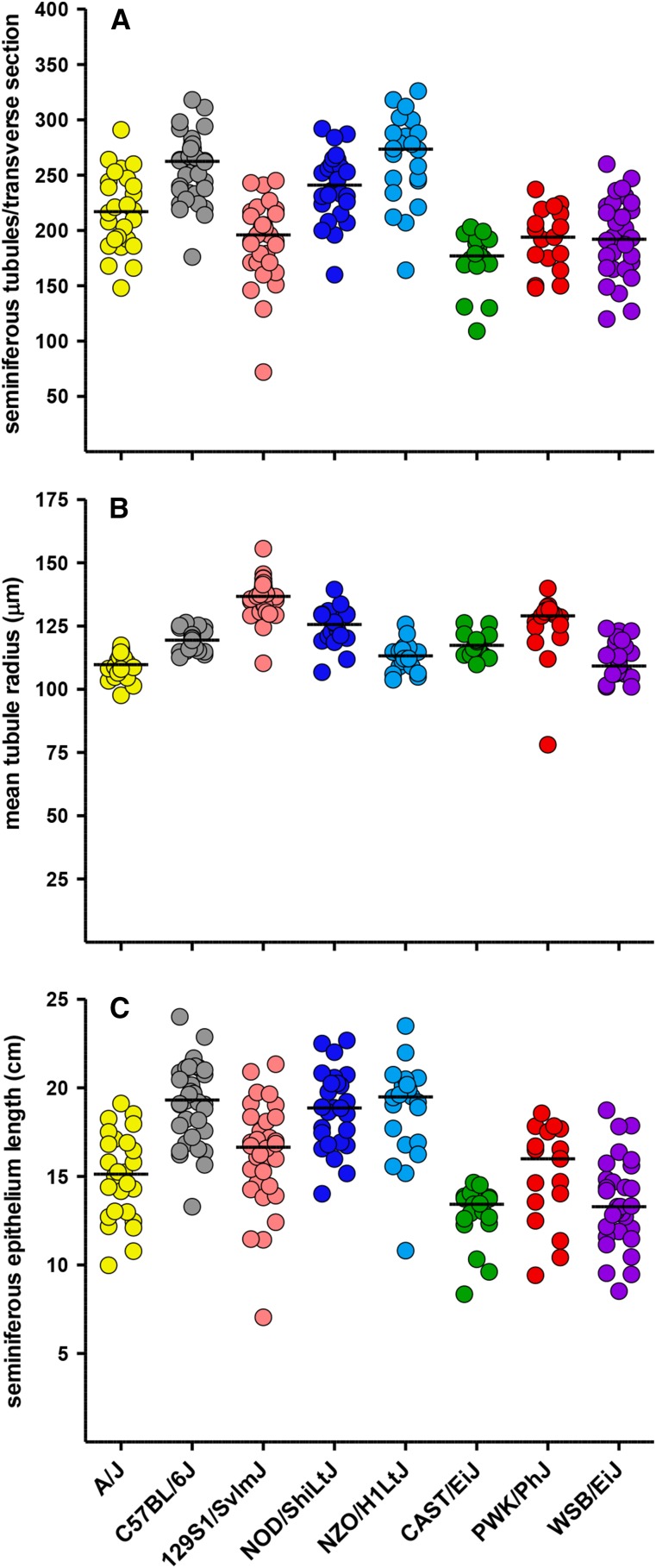
Number and dimensions of seminiferous tubules. Automated software developed for this project was used to identify, count, and measure the radii of seminiferous tubule cross sections in a composite image of a complete transverse section near the testis midline. The graphs show the number of (A) tubule cross sections, (B) the mean tubule radius, and the (C) calculated length of the seminiferous epithelium in each transverse section. Seminiferous epithelium length = mean tubule radius × 2 × π × number of tubule cross sections.

The number of seminiferous tubule cross sections per transverse testis image had a heritability of 0.5 (Table 1) and was not correlated with age (Table S5). C57BL/6J and NZO/H1LtJ mice had significantly more tubule cross sections, whereas CAST/EiJ mice had significantly fewer tubule cross sections than other strains (Figure 3A; Table S3). This trait was correlated with body weight, reproductive organ weights, and sperm counts (Table S4).

We estimated the mean radius of the tubules in each testis transverse section (Figure 3B). This parameter was highly heritable (0.57; Table 1) and varied significantly among the CC founder strains with A/J, NZO/HILtJ, and WSB/EiJ males having small average radii (Table S3) and 129S1/SvImJ, NOD/ShiLtJ, and PWK/PhJ having large average radii (Table S3). We then used the mean tubule radius and the number of tubules to estimate the length of the seminiferous epithelium in each transverse section (Figure 3C). The heritability for this trait was 0.48 and was significantly greater in C57BL/6J and NZO/H1LtJ mice and significantly lower in CAST/EiJ and WSB/EiJ mice.

**Figure 4:**
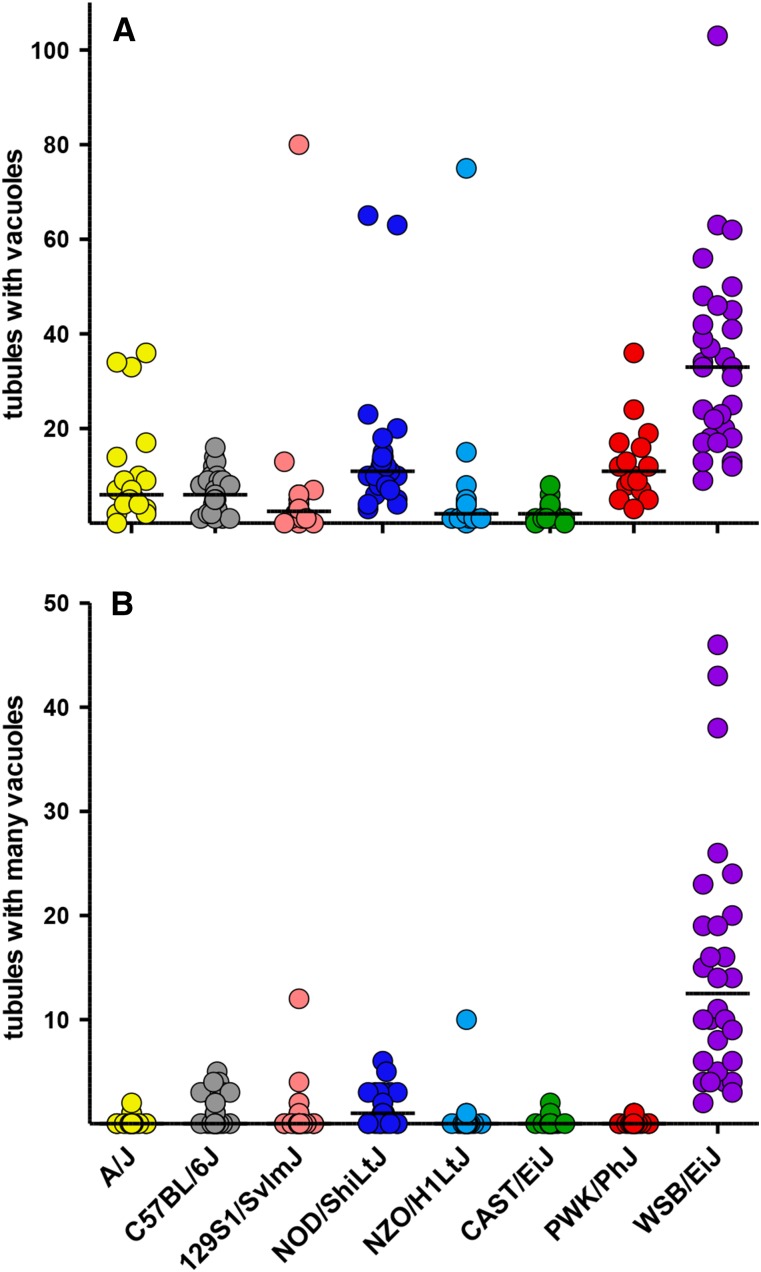
Frequency of vacuoles in seminiferous tubules. In the composite testis images, the number of (A) tubules with vacuoles and (B) tubules with many vacuoles were counted.

The presence of vacuoles in tubule cross sections was highly variable among the eight founder strains (Figure 4A; Table S3), and had a heritability of 0.35 (Table 1). Males from both NOD/ShiLtJ and WSB/EiJ strains had significantly more vacuoles, whereas males from 129S1/SvImJ, NZO/H1LtJ, and CAST/EiJ strains had fewer vacuoles (Table S3). When only tubules with many vacuoles were counted (Figure 4B), the heritability increased to 0.39 (Table 1) and the only outlier was the WSB/EiJ strain (Table S3). Interestingly, the number of tubules with vacuoles is positively correlated with age, but the significance of the age effect is dramatically reduced for the more severe phenotype (Table S5). There was also evidence of age by strain interaction.

**Figure 5:**
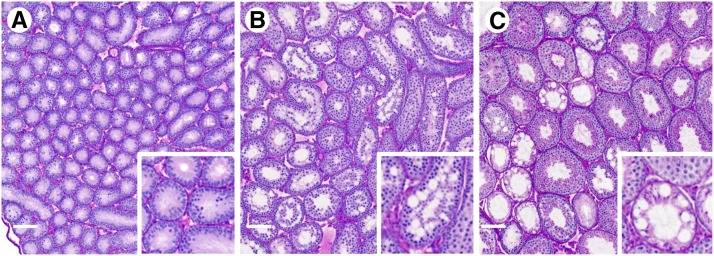
Testis histology of juvenile WSB/EiJ males. Testes from WSB/EiJ males between 2 and 6 wk of age were examined to determine the onset of the vacuole phenotype. Although vacuoles were rarely seen in the seminiferous epithelium at (A) 2 wk of age, extensive vacuolization was apparent at (B) 3 and (C) 6 wk of age. These images are shown at the same magnification (white bars, lower left = 100 μm) to illustrate the increase in tubule diameter and appearance of the lumen that occurs during this interval. Greater magnification inserts highlight the histologic features of individual tubules.

To determine the developmental timing of this previously unreported phenotype, we examined composite images from 2-, 3-, and 5- to 6-wk-old WSB/EiJ and C57BL/6J mice, when successive stages of spermatogenic cells are first appearing in the testis (Bellvé *et al.* 1977). Vacuoles were scored in testes from at least three mice at each age. Few vacuoles were seen at 2 wk of age in either strain (Figure 5A, Figure S3A), when spermatogonia and spermatocytes are the only germ cells present and lumen formation is beginning in some tubules. In contrast, tubules with many vacuoles were common in WSB/EiJ mice at 3 wk of age when round spermatids begin to appear (Figure 5B) and at 5−6 wk of age when elongating spermatids are evident (Figure 5C). Vacuoles were not apparent in 3-wk-old C57BL/6J mice (Figure S3B).

The number of tubules with germ cell loss had low heritability (0.15; Table 1), with the greatest number of affected tubules in the WSB/EiJ strain (Figure S4A; Table S3). There is a minor effect of age on germ cell loss (Table S5). Germ cell loss was significantly lower in 129S1/SvImJ and CAST/EiJ testes (Table S3). Other testicular defects also had low heritability (Table 1). Tubules with abnormal germ cells were most frequently observed in NZO/H1LtJ testes (median = 12.5 tubules) compared with other strains (median = 0−6 tubules), although the mean number of tubules with abnormal germ cells was comparable in A/J (15.35), NOD/ShiLtJ (13.12), and NZO/H1LtJ (15.91) testes (Figure S4B; Table S3). Tubules with germ cell sloughing were rare in all strains. NZO/H1LtJ testes had the greatest mean number of tubules with germ cell sloughing (10.05), although a few mice in all strains except NOD/ShiLtJ had >10 tubules with this defect (Table S1).

### Sperm counts

**Figure 6:**
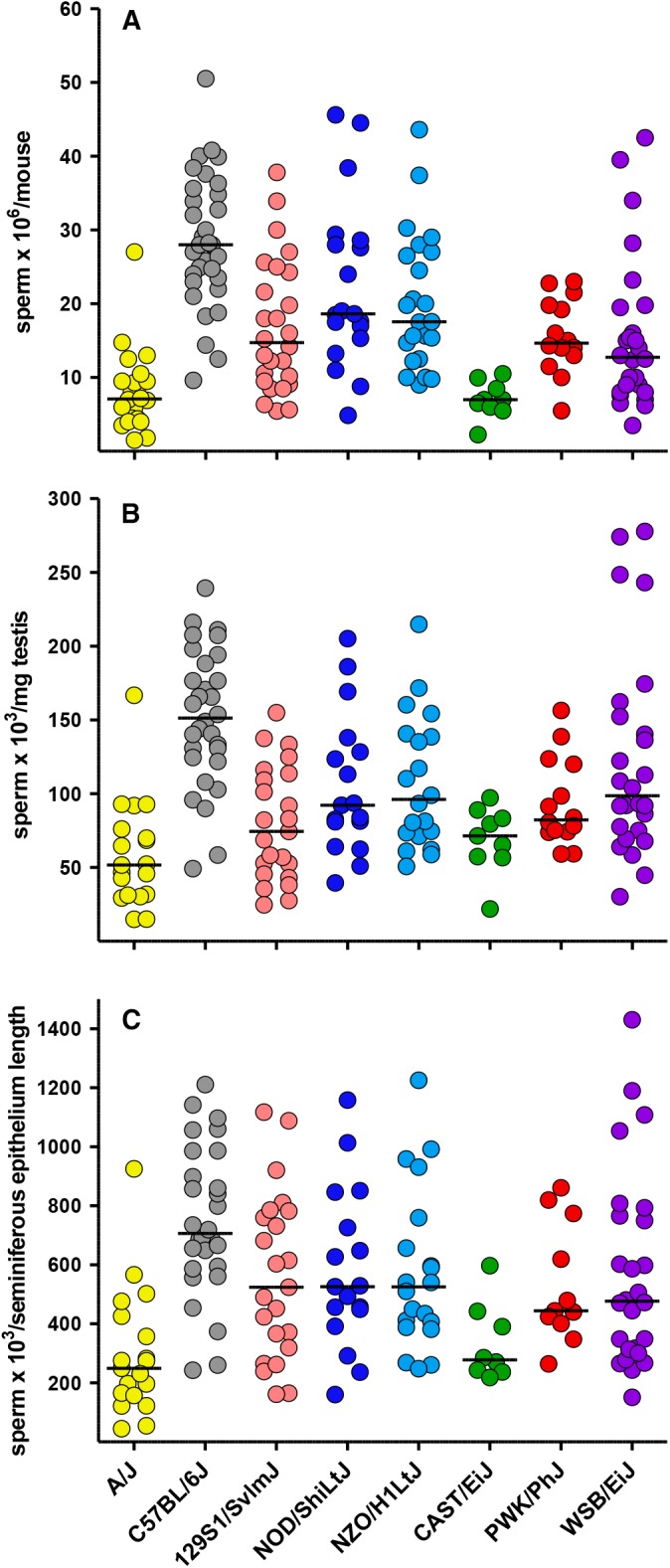
Sperm counts. Sperm from the right cauda epididymis were counted using a hemocytometer and graphed to show (A) the total sperm count × 10^6^ per mouse, (B) the number of sperm × 10^3^ produced per mg of testis, and (C) the number of sperm × 10^3^ produced per length of the seminiferous tubule epithelium in a midline transverse testis section.

By the use of a procedure to maximize recovery, sperm were collected from the right cauda epididymis of each animal and counted. Sperm counts/mouse (Figure 6A) had lower heritability (0.38; Table 1) than reproductive organ weights, and the heritability decreased when sperm counts were normalized for testis weight (Figure 6B) or the length of the seminiferous epithelium per transverse cross section (Figure 6C). Overall, there are outliers for both high (C57BL/6J) and low sperm counts (A/J and CAST/EiJ; Table S3). Although normalization for testis weight and length of the seminiferous epithelium had some impact on the value of the *z* score, the strains with high and low traits values remain unchanged (Table S3). Sperm counts were positively correlated with both testis and E+V weights (Table S4) but showed substantial within strain variation. Sperm counts were not correlated with age (Table S4 and Table S5).

### Sperm morphology

**Figure 7:**
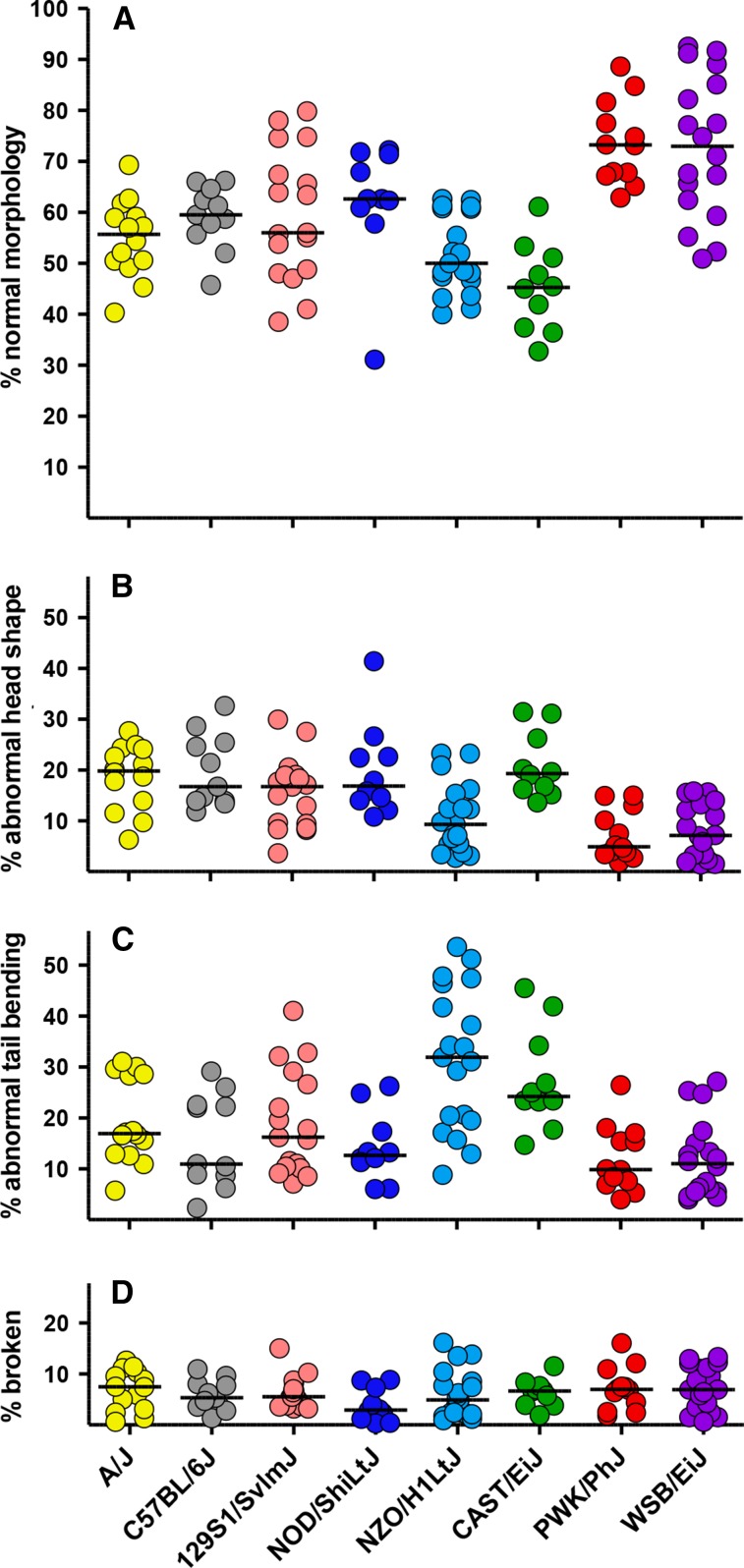
Sperm morphology. Fixed sperm were examined by phase contrast microscopy and counted to determine the percentage with (A) normal morphology, (B) significant defects in the morphology of the sperm head, (C) abnormal bends in the tail that were ≥90°, or (D) breaks that detach the tail from the sperm head or sever the tail in more distal locations.

To assess sperm quality, both morphology and motility were examined after gentle release of sperm from the left cauda epididymis into HTF medium. Sperm morphology was scored after fixation and labeling with fluorescent peanut agglutinin lectin to confirm the retention of intact acrosomes. The percentages of sperm with normal morphology, abnormal head shape, abnormal tail bending, and broken tails were determined (Figure 7). The first three classes had moderate heritability (0.34−0.47, Table 1), whereas broken tails had very low heritability (0.07; Table 1) indicating that this trait is driven mostly by environmental and/or procedural factors. PWK/PhJ and WSB/EiJ mice had the greatest percentages of sperm with normal morphology (Figure 7A), with median percentages of normal sperm above 70% for both strains. These values were less variable for PWK/PhJ sperm, resulting in a significantly greater *z* score (Table S3). The percentages of normal sperm morphology in both NZO/H1LtJ and CAST/EiJ mice were significantly lower than other strains (Table S3). Four strains had greater percentages of abnormal head shape (A/J, C57BL/6J, 129S1/SvImJ, and NOD/ShiLtJ; Figure 7B); NZO/H1LtJ had the greatest percentage of abnormal tail bending (≥90°; Figure 3C), and CAST/EiJ had higher percentages of both abnormalities (Figure 7, B and C). Similar numbers of broken tails (median ≤7.5%), severed at the head/neck junction or at more distal locations along the length of the flagellum, were observed in all strains (Figure 7D). The percentage of sperm with normal morphology was not correlated with age, but the percentages of sperm with abnormal head shape, abnormal tail bending and broken tails were positively correlated with age (Table S4 and Table S5).

### Sperm motility

**Figure 8:**
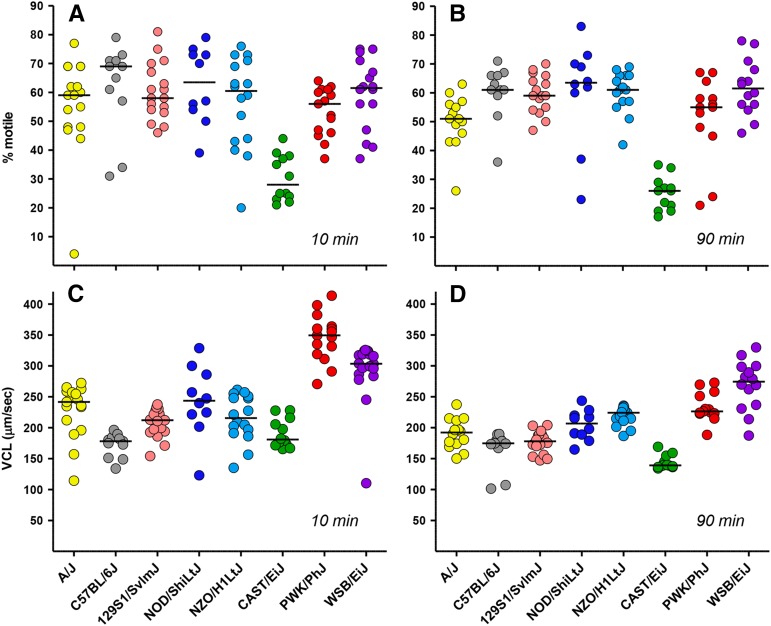
Sperm motility. Computer-assisted sperm analysis was used to determine the percentage of motile sperm, curvilinear velocities (VCLs), and other motility parameters (Table S1 and Table S2) during *in vitro* capacitation. The percentage of motile sperm are shown for (A) the initial time point and (B) after incubation for 90 min. VCLs (μm/sec) are also shown for the (C) initial and (D) 90-min time points.

CASA was used for the quantitative assessment of motility throughout a 2-hr incubation period under conditions that support sperm capacitation. Multiple physiological changes required for fertilization occur during capacitation, including the development of hyperactivated motility, which typically reaches maximum levels by 90 min (Goodson *et al.* 2011). The percentage of motile sperm is a moderately heritable trait in the eight inbred strains (Table 1), with heritability higher at 90 min (0.60) than at the initial 10 min time point (0.42). The percentage of motile sperm at 10 min was significantly greater for C57BL/6J mice than all other strains (Figure 8A; Table S3). In all five classical inbred strains and in PWK/PhJ and WSB/EiJ mice, mean and median percentages of motile sperm were ≥50% initially (Figure 8A) and after 90 min (Figure 8B). In contrast, the percentage of motile sperm in CAST/EiJ mice remained ≤30%, significantly lower than all other strains at both time points (Table S3).

Mean VCL, which measures the average velocity over point-to-point CASA tracks, had high heritability (0.68−0.71) at both 10 and 90 min (Table 1). Sperm from PWK/PhJ and WSB/EiJ mice were significantly faster (median VCL >300 µm/sec) than sperm from all other strains at the initial time point (Figure 8C; Table S3). Sperm from these strains and NZO/H1LtJ had significantly greater VCL after *in vitro* capacitation for 90 min (Figure 8D; Table S3). C57BL/6J and CAST/EiJ sperm had significantly lower VCL (median <185 μm/sec) at both time points, along with 129S1/SvImJ sperm at 90 min.

CASA determines VCL and other kinematic parameters of sperm motility (Table S1 and Table S2), but does not directly identify hyperactivation or other changes in motility that occur during capacitation. We used CASAnova, a support vector machines program (Goodson *et al.* 2011), to compare physiologically relevant motility patterns between strains. This automated program classifies individual CASA sperm tracks as progressive, intermediate, hyperactivated, slow, or weakly motile. For comparison, motility analyses of sperm from CD1 mice (Goodson *et al.* 2011) classified >75% of motile sperm as progressive immediately after isolation. Hyperactivated motility of CD1 sperm increased during capacitation reaching maximal levels of ∼20–30% by 90 min, and the percentage of nonvigorous motility patterns (slow and weakly motile) also increased during this 2-hr interval.

**Figure 9:**
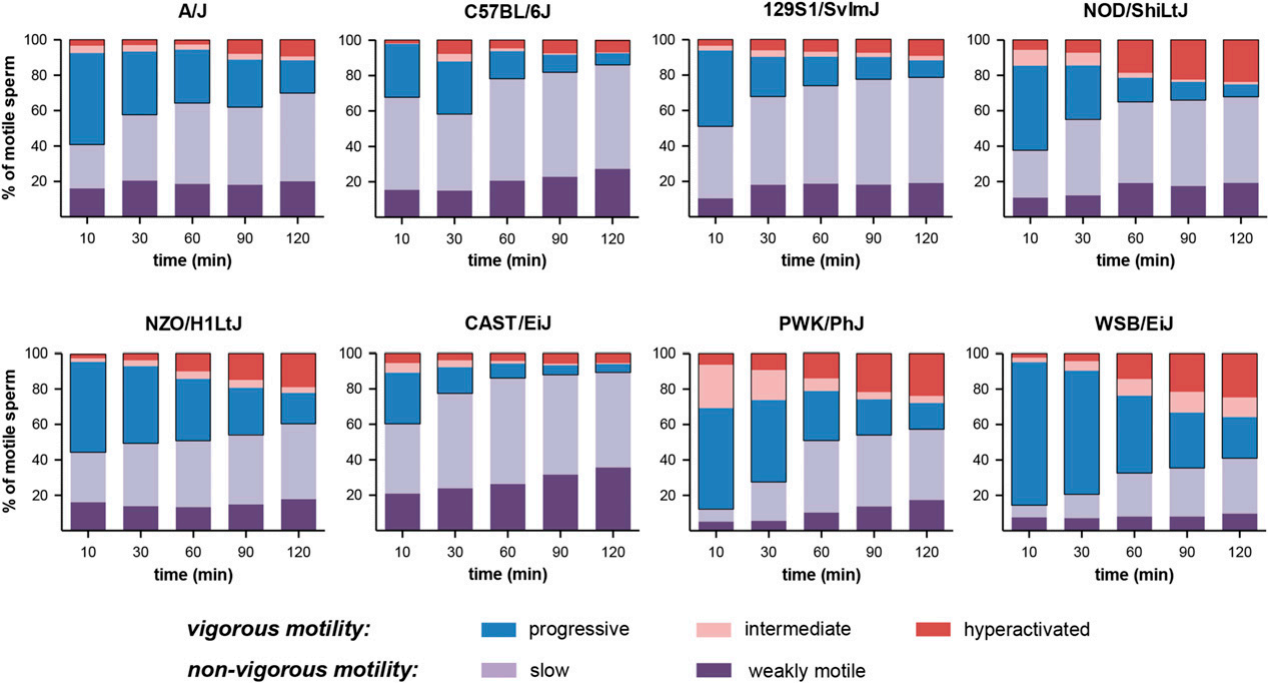
Motility profiles during *in vitro* capacitation. CASAnova was used to quantitate five motility patterns at 30-min intervals throughout capacitation. At each time point, the stacked bar shows the percentage of sperm that were classified as progressive (blue), intermediate (pink), hyperactivated (red), slow (lavender), and weakly motile (purple). Vigorous motility is the sum of sperm that were progressive, intermediate and hyperactivated (outlined in black). The percentage of hyperactivated sperm typically increases during capacitation, along with a decrease in progressive motility patterns and an increase in nonvigorous (slow and weakly motile) patterns.

CASAnova was used to monitor motility patterns at 30 min intervals throughout *in vitro* capacitation. The mean percentages of the five motility patterns were calculated for each strain at each time point and are shown as stacked bar graphs in Figure 9. The progressive (blue), intermediate (pink), and hyperactivated (red) segments of each bar are outlined in black to indicate the sum of the vigorous motility categories. At the initial time point, the mean percentages of vigorous motility were significantly greater (>85%) for PWK/PhJ and WSB/EiJ sperm and significantly lower (<40%) for C57BL/6J and CAST/EiJ sperm (Table S3). Sperm from PWK/PhJ, WSB/EiJ, and NZO/H1LtJ mice maintained the greatest mean percentages of vigorous motility (>40%) throughout the 2-hr capacitation period. At 90 min, the mean percentages of vigorous motility were significantly greater for NZO/H1LtJ and WSB/EiJ sperm and significantly lower for C57BL/6J, 129S1/SvImJ, and CAST/EiJ sperm (Table S3). Hyperactivated motility patterns increased with time in sperm from all strains except CAST/EiJ (Figure 9), with the greatest mean percentages for PWK/PhJ, WSB/EiJ, NOD/ShiLtJ, and NZO/H1LtJ sperm (15–25% at 90−120 min). Based on *z* scores, the percentages of hyperactivated sperm at 90 min were significantly greater in NOD/ShiLtJ and PWK/PhJ mice and significantly lower in A/J and CAST/EiJ mice.

Most motility parameters monitored in this study were not correlated with age (Table S5), but VCL and the percentage of hyperactived sperm at 90 min have a moderate age effect and age by strain interaction (Table S5).

### Lactate production

**Figure 10:**
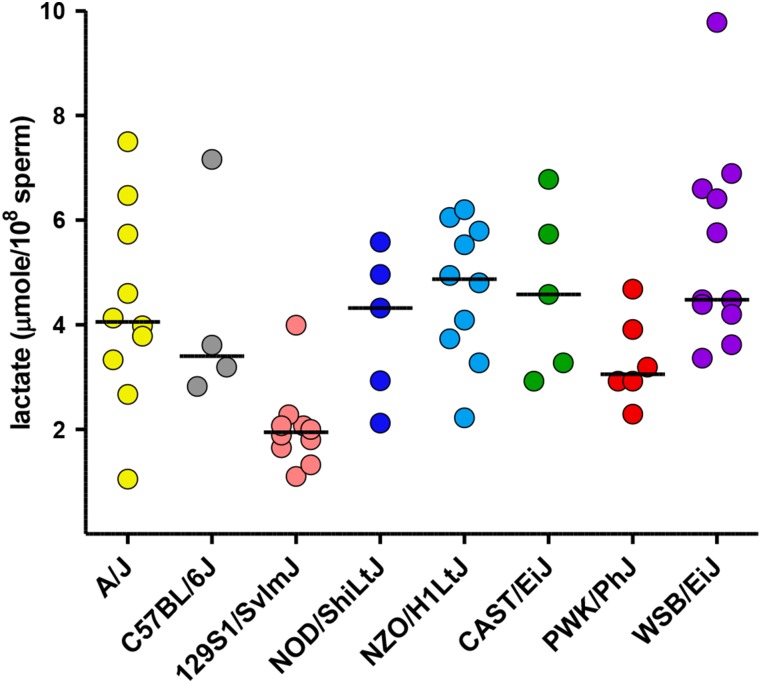
Lactate production. A spectrophotometric assay was used to monitor sperm lactate production and accumulation in the medium during *in vitro* capacitation. Lactate production (μmole/10^8^ sperm) serves as an indicator of sperm glycolytic activity.

The glycolytic pathway has several unique isozymes in mammalian sperm and produces ATP that is essential for sperm motility and male fertility (Miki *et al.* 2004; Odet *et al.* 2008; Danshina *et al.* 2010; Goodson *et al.* 2012). As an initial assessment of potential strain differences in sperm metabolism, we monitored lactate accumulation in the medium during *in vitro* capacitation, an endpoint long used as an indicator of glycolytic activity (Mann and Lutwak-Mann 1981). Lactate production had moderate heritability (0.29, Table 1) and was not correlated with age (Table S5). Sperm from six strains produced comparable levels of lactate during the 2-hr incubation period (mean >4 μmole/10^8^ sperm; Figure 10). In contrast, mean lactate production was low for PWK/PhJ sperm and very low for 129S1/SvImJ sperm (Table S3).

## Discussion

Strains surveys for biomedical traits provide a simple and efficient avenue to determine the variability of those traits and estimate broad sense heritability and variation due to environmental factors and measurement error. In addition, these surveys allow integration of data over a wide phenotypic space and assessment of correlation between multiple traits. Finally, inbred strains can be used to explore the developmental basis of a trait and the effects of many types of intervention. The Mouse Phenome Database provides testis and seminal vesicle weights and sperm counts for 14 inbred strains and a variety of males from early generations of inbreeding of the CC. The data reported here increase the number of CC founder strains with the addition of NZO/HILtJ and PWK/PhJ, the number of mice analyzed per strain (from five to an average of 21 per trait), the age range and, most importantly, the number of phenotypes. Specifically, we provide the first strain survey for testis histology and for multiple parameters of sperm quality, including motility, morphology and glycolytic production of lactate.

Reproductive organ weights and sperm counts in this study were consistent with those reported in the Mouse Phenome Database for 8- to 10-wk-old mice. Seminal vesicle weights, which were highly correlated with age and body weight (Table S4 and Table S5), were greater in our survey of mice with a broader age range. Our sperm counts also were greater for most strains, and our rigorous sperm collection method may have contributed to these differences.

The collection of a comprehensive set of male reproductive phenotypes provides a nuanced picture of reproductive fitness of individual strains. As in the infertility clinic (Isidori *et al.* 2005), deficiencies in sperm count, morphology, and/or motility are associated with reduced male fertility in mouse knockout models (Matzuk and Lamb 2008), hybrid sterility models (Storchova *et al.* 2004; Oka *et al.* 2004; Good *et al.* 2008; White *et al.* 2011, 2012), and in natural hybrids (Turner *et al.* 2012). All three of these measures of sperm quality were consistently low in the wild-derived CAST/EiJ strain, noted as a “challenging breeder” by the Jackson Laboratory (http://jaxmice.jax.org/strain/000928.html). Among classical inbred strains, A/J males also had low sperm counts, but average morphology and motility. None of the CC founder strains had advantageous values for all sperm traits. C57BL/6J males had the greatest sperm counts, but less vigorous motility. The wild-derived PWK/PhJ and WSB/EiJ strains had the greatest percentage of normal sperm morphology and vigorous motility, with average sperm counts. These differences exemplify the limitations of using a single mouse strain as “normal” or “wild-type” controls in biomedical studies.

We hypothesized that if sperm count is, as expected, directly correlated to the total area of the seminiferous epithelium lining the tubules, then the best predictor of sperm counts in our survey should be the length of the seminiferous epithelium per transverse section. Figure 6C and Table S4 show that the variance in sperm counts is reduced after accounting for length of the seminiferous epithelium per transverse section, supporting this hypothesis. The sperm counts in CAST/EiJ and A/J mice remained low after this correction, suggesting the presence of additional causes.

The genetic regulation of motility and its potential contribution to hybrid sterility have not been widely studied. Multiple QTL for sperm count and morphology have been identified in analyses of hybrids between *Mus musculus* subspecies (Storchova *et al.* 2004; Oka *et al.* 2004; Good *et al.* 2008; White *et al.* 2011; 2012). In contrast, few motility-associated QTL have been identified even though differences in motility between hybrids have been reported (Oka *et al.* 2004; Turner *et al.* 2012). A study of RI strains derived from KE and CBA/Kw mice (likely to represent different subspecies of house mice) identified one motility-associated QTL for beat cross frequency, a CASA motility parameter monitoring the frequency that the sperm head crosses the average path (Golas *et al.* 2004). There has also been progress in identifying genes responsible for motility defects in *t*-haplotype mice (Herrmann *et al.* 1999; Bauer *et al.* 2012) and knockout models (Matzuk and Lamb 2008). Our studies found that multiple measures of sperm motility vary between the CC founder strains and are highly heritable. The percentage of motile sperm was typically above 50% throughout the 2-hr time course for all strains except CAST/EiJ, which had very low values ≤30%. There was greater variation between strains in sperm velocity and the patterns of motility observed during *in vitro* capacitation. PWK/PhJ and WSB/EiJ sperm had very high VCL. Motility profiles for these strains and NZO/HILtJ (Figure 9) were most similar to outbred CD1 mice (Goodson *et al.* 2011), maintaining ≥40% vigorous motility throughout the time course with 15–25% of motile sperm exhibiting hyperactivation. NOD/ShiLtJ sperm also achieved comparable levels of hyperactivated motility. By 90 min, VCL and vigorous motility dropped to significantly lower levels for C57BL/6J, 129S1/SvImJ and CAST/EiJ sperm.

Since glycolysis is required for normal sperm motility (Miki *et al.* 2004; Odet *et al.* 2008; Danshina *et al.* 2010; Goodson *et al.* 2012), we measured lactate production via this pathway during *in vitro* capacitation. Previous studies demonstrated that phosphoglycerate kinase 2, one of the sperm-specific glycolytic enzymes, has very low activity in 129 strains (VandeBerg *et al.* 1976; VandeBerg and Blohm 1977; Eicher *et al.* 1978) compared with A/J, C57BL/6J, and several other strains. Lactate production was very low in 129S1/SvImJ sperm, as expected, and also low in PWK/PhJ sperm. In both strains the percentage of motile sperm remained greater than 50% throughout *in vitro* capacitation. VCL was reduced in 129S1/SvImJ sperm by the 90-min time point but remained high in PWK/PhJ sperm. Sperm from other strains produced comparable amounts of lactate, suggesting that the poor motility observed in CAST/EiJ sperm is not correlated with a defect in glycolysis.

Early studies with C57BL/6 reported age-related deficits in male fertility, sperm number, and sperm quality in mice >20 mo old (Franks and Payne 1970; Parkening *et al.* 1988). We did not observe a correlation between age and testis weight, sperm count, or several characteristics of sperm motility (Table S4 and Table S5), indicating that all CC founder strains maintain sperm production and standard measures of sperm motility until at least until 16 mo of age. The percentage of sperm with normal morphology was not correlated with age. However, the percentage of sperm with abnormal morphology did show an increase with age and the percent of abnormal tail bending showed an interaction between age and strain (Table S5). In this study we did not monitor paternal age effects on fertility, which may decline without changes in these parameters of sperm quality, as seen in a recent study of outbred CF1 mice ≥12 mo of age (Katz-Jaffe *et al.* 2013).

Our analysis of testis histology reveals that the number of tubules per testis cross-section is variable and heritable. Very little is known about the developmental and genetic regulation of this process. The most notable defect we observed in testis histology was the presence of a large number of vacuoles within seminiferous tubule cross sections in the WSB/EiJ strain. Vacuoles were present in an average of 15% of tubule cross sections in WSB/EiJ males and a third of those have many vacuoles. To our knowledge, this phenotype has not been reported previously and is unique to this wild-derived strain among the CC founders. Vacuolization is considered an indicator of potential disturbance of Sertoli cell function in testicular toxicology analyses (Lanning *et al.* 2002). Vacuoles in the seminiferous epithelium also have been noted as a consequence of aging in both mice (Takano and Abe 1987; Tanemura *et al.* 1993) and men (Paniagua *et al.* 1991). In this study, both the presence of vacuoles and the many vacuole phenotype (observed in WSB/EiJ males) were correlated with age (Table S5). However, tubules with many vacuoles were readily observed in WSB/EiJ testes at 3 wk of age but not at 2 wk. During this interval, Sertoli cell junctions are assembled to form the blood-testis barrier (Nagano and Suzuki 1976; Meng *et al.* 2005), contributing to lumen formation that is apparent by 3 wk of age (Pace *et al.* 2000). The temporal correlation of vacuole appearance with blood-testis barrier formation again suggests that defects in Sertoli cell function may contribute to this abnormal phenotype.

The WSB/EIJ strain is commonly used as the reference genome for the *Mus musculus domesticus* subspecies and in crosses to study the genetics of speciation and of male reproductive traits (Dumont *et al.* 2011; White *et al.* 2011, 2012). Regarding the CC, the vacuolization phenotype *per se* should not be the cause for extinction, as the WSB/EiJ males are able to reproduce. However, WSB/EiJ males have advantageous characteristics for many other key reproductive traits, such as low number of tubules with abnormal germ cells, low number of sperm with abnormal head shape, and high VCL and percent of vigorous sperm motility at 10 and 90 min. We speculate that during the generation of the CC RI lines the WSB/EiJ alleles responsible for vacuolization will be combined with deleterious alleles for sperm motility, hyperactivation and/or sperm number from the other CC founders and that the accumulation of multiple hits in male reproductive parameters will severely reduce fertility and increase extinction. Moreover, we have scored the vacuolization phenotype in 47 F1 hybrids between WSB/EiJ and the other seven founder strains (Figure S5, see also histology website). The vacuolization phenotype is significantly different dependent on the genetics background (*P* < 0.0001, one-way ANOVA). Interestingly, the phenotype is suppressed in crosses to five different backgrounds, it is present at low levels in F1 hybrids to CAST/EiJ, and, most importantly, it is enhanced in the F1 hybrids to PWK/PhJ (Figure S5). We note that the phenotype is also significantly different depending on background (*P* < 0.001, one-way ANOVA) after excluding the PWK/PhJ F1 hybrids. In conclusion, crosses within the same subspecies suppress the vacuolization phenotype but crosses to other subspecies may enhance it. This evidence of a role of dominance and epistasis in deleterious reproductive traits fits very well with the prediction that genetic incompatibilities at multiple loci drive extinction in the CC.

A key goal of this study was to determine the heritability of male reproductive traits in the eight founders of the CC as a steppingstone to QTL mapping in the CC population. Although in our survey the heritability is high for many of these traits, we expect that genetic mapping in the CC RI lines will be challenging due to both the quantitative nature of the traits and the likely presence of epistatic interactions between alleles derived from different subspecies. Interactions between species-specific alleles are predicted to reduce reproductive fitness (Turner *et al.* 2012). Mapping these epistatic loci may be challenging due to lack of statistical power but it has been achieved for other biomedical traits using specific CC RI lines (Rogala *et al.* 2014).

## Supplementary Material

Supporting Information
