# Supplementary material for: The Founder Strains of the Collaborative Cross Express a Complex Combination of Advantageous and Deleterious Traits for Male Reproduction
Source: G3 (Bethesda). 2015 Oct 13;5(12):2671–83. doi: 10.1534/g3.115.020172 (PMC4683640; doi:10.1534/g3.115.020172)
Supplement: Supporting Information [file supp_5_12_2671_v2_index.html]

The Founder Strains of the Collaborative Cross Express a Complex Combination of Advantageous and Deleterious Traits for Male Reproduction — Supporting Information 

# The Founder Strains of the Collaborative Cross Express a Complex Combination of Advantageous and Deleterious Traits for Male Reproduction

## Supporting Information for Odet *et al.*, 2015

**Files in this Data Supplement:**

- Figure S1 - Age of mice. (.zip, 71 KB)
- Figure S2 - A screen capture from the image analysis tool for annotating testis histology. (.zip, 1,685 KB)
- Figure S3 - Testis histology of juvenile C57BL/6J males. (.zip, 3,174 KB)
- Figure S4 - Number of seminiferous tubules with germ cell loss and abnormal germ cells. (.zip, 103 KB)
- Figure S5 - Frequency of vacuoles in seminiferous tubules in F1 hybrids involving the WSB/EiJ strain. (.zip, 89 KB)
- Table S1 - All primary data for 248 mice. (.zip, 81 KB)
- Table S2 - All primary motility data for 116 mice. (.zip, 145 KB)
- Table S3 - Means, median and z scores for traits in Table 1. (.zip, 25 KB)
- Table S4 - Correlations between traits shown in Table 1. (.zip, 79 KB)
- Table S5 - P values for two-way ANOVA with age, strain and interaction between both. (.zip, 75 KB)
