# Supplementary material for: The Founder Strains of the Collaborative Cross Express a Complex Combination of Advantageous and Deleterious Traits for Male Reproduction
Source: G3 (Bethesda). 2015 Oct 13;5(12):2671–83. doi: 10.1534/g3.115.020172 (PMC4683640; doi:10.1534/g3.115.020172)

Figure S1. Age of mice. Mice used in this study were between 70 and 461 days of age, with a similar distribution for each strain. The colors represent the eight founder strains as follows: A/J, yellow; C57BL/6J, grey; 129S1/SvImJ, pink; NOD/ShiLtJ, dark blue; NZO/H1LtJ, light blue; CAST/EiJ, green; PWK/PhJ, red; and WSB/EiJ, purple. Each male is represented by a circle.

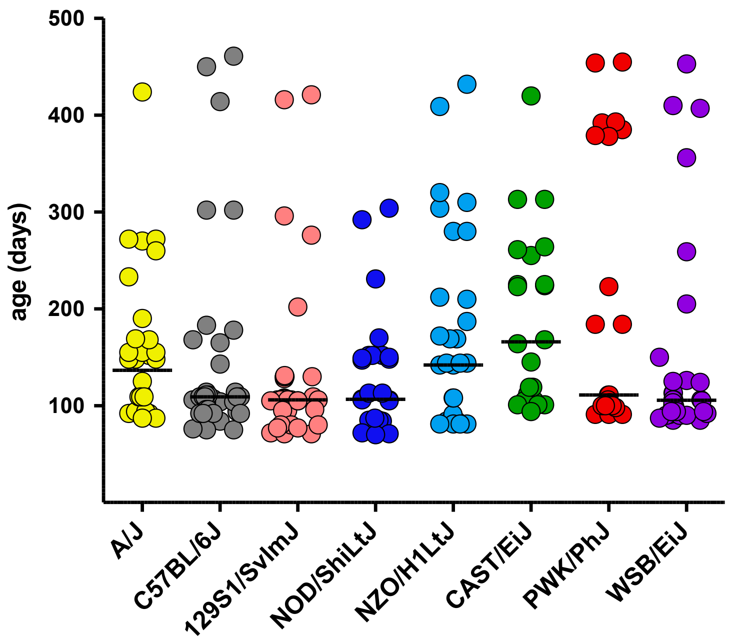

Supplement: Supporting Information [file supp_g3.115.020172_FigureS1.zip › FigureS1.pdf]
