# Supplementary material for: The Founder Strains of the Collaborative Cross Express a Complex Combination of Advantageous and Deleterious Traits for Male Reproduction
Source: G3 (Bethesda). 2015 Oct 13;5(12):2671–83. doi: 10.1534/g3.115.020172 (PMC4683640; doi:10.1534/g3.115.020172)

Figure S2. A screen capture from the image analysis tool for annotating testis histology. A composite testis image from a PWK/PhJ male (GG1804) is shown on the right, with glyphs denoting the centers of seminiferous tubules and other features. A magnifier inset showing the full resolution histology image under the user's cursor is shown in the upper left. The shape of each glyph indicates whether the structure is a seminiferous tubule (± abnormal germ cells) or other feature such as a blood vessel or part of the rete testis. The color of the glyph indicates the germ cell composition of each tubule. Other histological features, such as the presence of vacuoles or sloughing of germ cells into the lumen, are annotated within the interior of the glyphs. The circles surrounding each glyph indicate the radius of the tubule's minor axis, with the color of the circle representing the tertile where the radius falls in the distribution for all tubules. This distribution is shown on left below the magnifier inset.

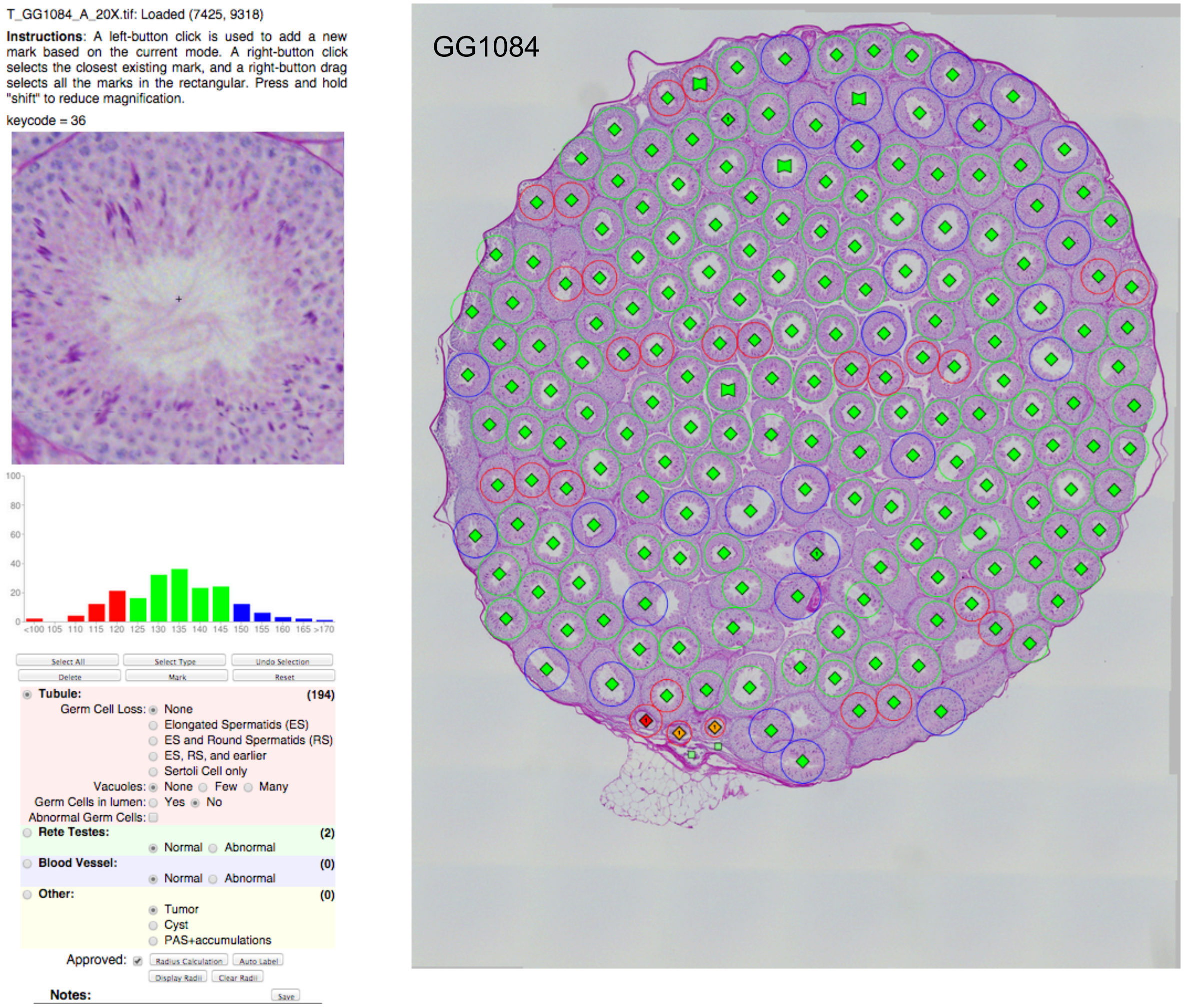

Supplement: Supporting Information [file supp_g3.115.020172_FigureS2.zip › FigureS2.pdf]
