# Supplementary material for: The Founder Strains of the Collaborative Cross Express a Complex Combination of Advantageous and Deleterious Traits for Male Reproduction
Source: G3 (Bethesda). 2015 Oct 13;5(12):2671–83. doi: 10.1534/g3.115.020172 (PMC4683640; doi:10.1534/g3.115.020172)

Figure S3. Testis histology of juvenile C57BL/6J males. Testes from C57BL/6J males at two weeks (A) and three weeks (B) of age are shown for comparison with WSB/EiJ juvenile males (Fig. 5). These images are shown at the same magnification (white bars, lower left = 100 μm) to illustrate the increase in tubule diameter and appearance of the lumen that occurs during this interval. Vacuoles in the seminiferous epithelium were rare at either age in C57BL/6J males.

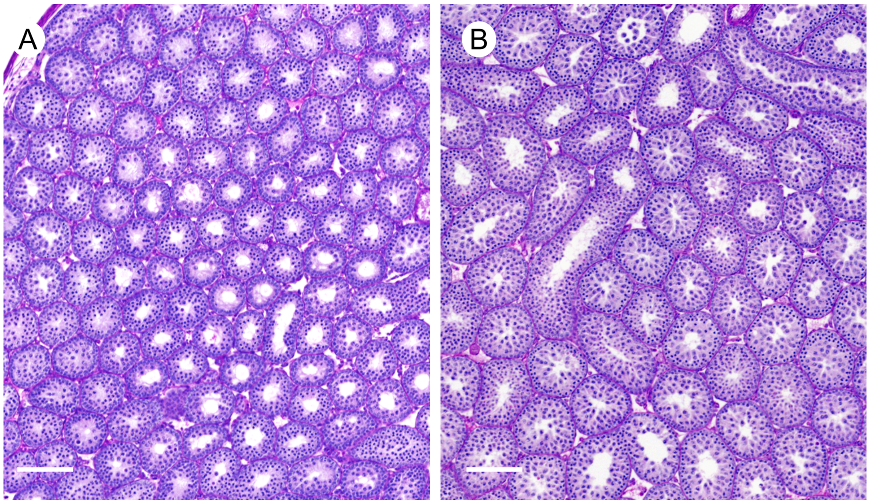

Supplement: Supporting Information [file supp_g3.115.020172_FigureS3.zip › FigureS3.pdf]
