# Supplementary material for: The Founder Strains of the Collaborative Cross Express a Complex Combination of Advantageous and Deleterious Traits for Male Reproduction
Source: G3 (Bethesda). 2015 Oct 13;5(12):2671–83. doi: 10.1534/g3.115.020172 (PMC4683640; doi:10.1534/g3.115.020172)

**Figure S4. Number of seminiferous tubules with germ cell loss and abnormal germ cells.** An image analysis tool was used to record and count abnormalities in each composite image of a transverse section near the testis midline. These graphs show the number of tubules with germ cell loss (A), which was correlated with the vacuole phenotype in WSB/EiJ males, and the number of tubules with abnormal germ cells (B).

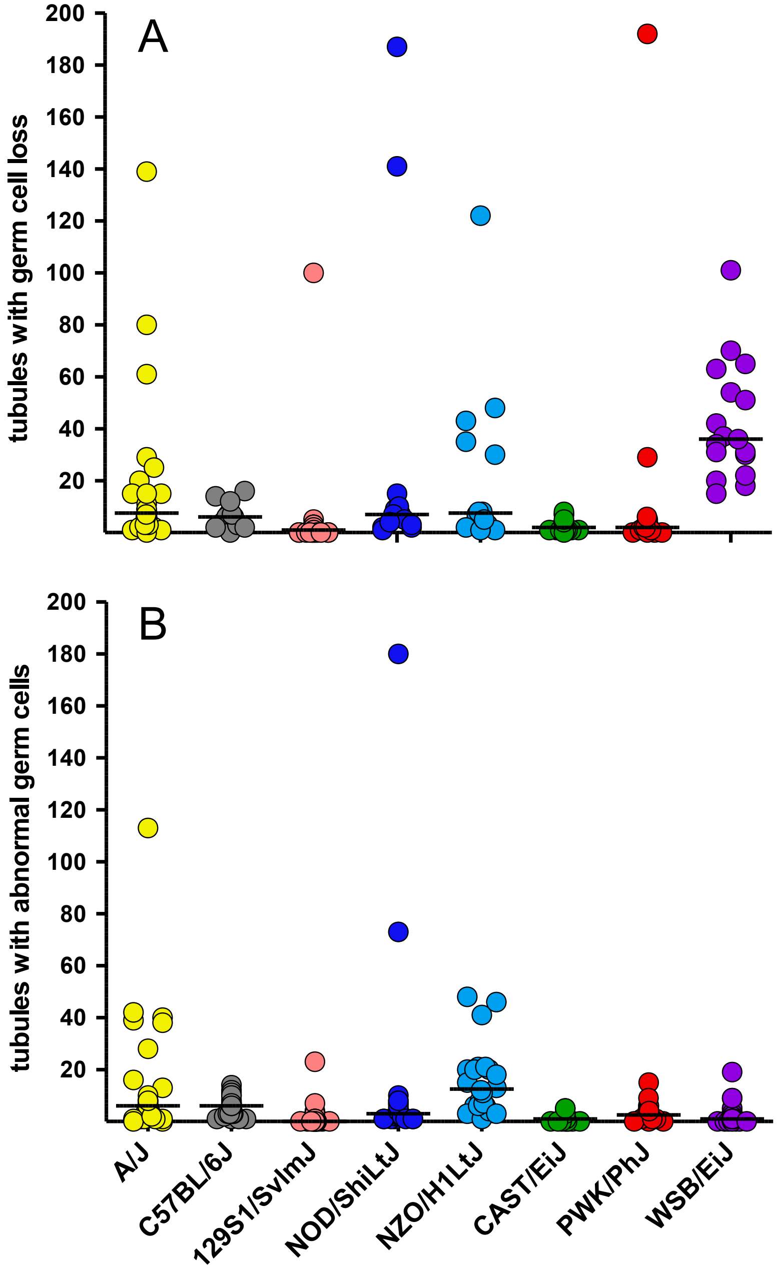

Supplement: Supporting Information [file supp_g3.115.020172_FigureS4.zip › FigureS4.pdf]
