# Supplementary material for: The Founder Strains of the Collaborative Cross Express a Complex Combination of Advantageous and Deleterious Traits for Male Reproduction
Source: G3 (Bethesda). 2015 Oct 13;5(12):2671–83. doi: 10.1534/g3.115.020172 (PMC4683640; doi:10.1534/g3.115.020172)

Figure S5. Frequency of vacuoles in seminiferous tubules in F1 hybrids involving the WSB/EiJ strain. Box plots for the number of tubules with vacuoles in 47 F1 hybrids between the WSB/EiJ strain and the other seven founders strains of the CC. The testis histology of the 47 samples can be found at http://database.csbio.unc.edu/Infertility.

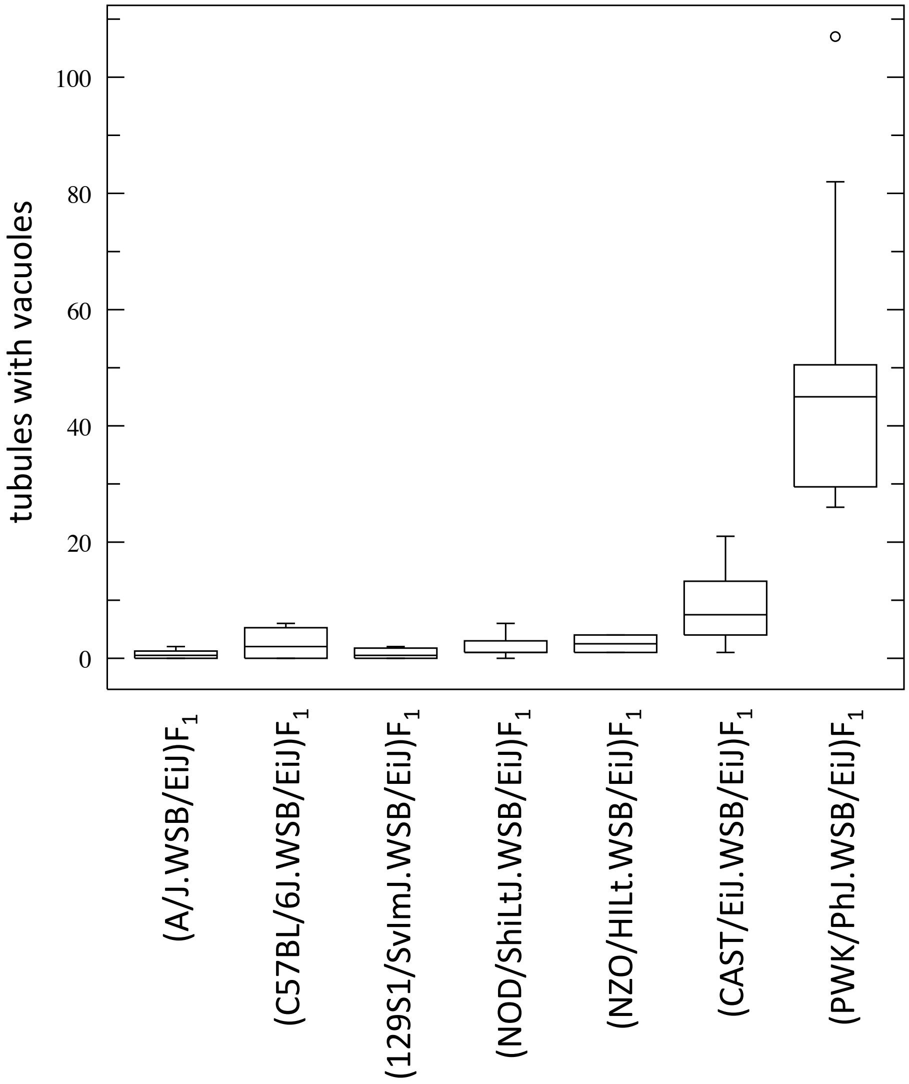

Supplement: Supporting Information [file supp_g3.115.020172_FigureS5.zip › FigureS5.pdf]
