# Supplementary material for: The Founder Strains of the Collaborative Cross Express a Complex Combination of Advantageous and Deleterious Traits for Male Reproduction
Source: G3 (Bethesda). 2015 Oct 13;5(12):2671–83. doi: 10.1534/g3.115.020172 (PMC4683640; doi:10.1534/g3.115.020172)
Supplement: Supporting Information [file supp_g3.115.020172_TableS4.zip › TableS4.pdf]

Table S4. Correlations between traits shown in Table 1.

	body weight	mean testis weight	mean epididymis + vas deferens weight	seminal vesicles weight	# of seminiferous tubules/transverse section	mean tubule radius	seminiferous epithelium length/transverse section	# of tubules with vacuoles	# of tubules with many vacuoles	# of tubules with germ cell loss	# of tubules with abnormal germ cells	# of tubules with germ cell sloughing
body weight	1.00	0.52	0.83	0.69	0.52	-0.03	0.49	-0.15	-0.17	0.08	0.19	0.28
mean testis weight	0.52	1.00	0.64	0.57	0.46	0.56	0.70	-0.34	-0.29	-0.35	-0.11	0.01
mean epididymis + vas deferens weight	0.83	0.64	1.00	0.72	0.60	0.08	0.63	-0.08	-0.09	0.13	0.15	0.23
seminal vesicles weight	0.69	0.57	0.72	1.00	0.47	0.11	0.51	-0.09	-0.16	0.04	0.09	-0.02
# of seminiferous tubules/transverse section	0.52	0.46	0.60	0.47	1.00	-0.21	0.92	-0.05	-0.09	0.15	0.20	0.13
mean tubule radius	-0.03	0.56	0.08	0.11	-0.21	1.00	0.18	-0.41	-0.31	-0.58	-0.27	-0.12
seminiferous epithelium length/transverse section	0.49	0.70	0.63	0.51	0.92	0.18	1.00	-0.21	-0.21	-0.09	0.08	0.08
# of tubules with vacuoles	-0.15	-0.34	-0.08	-0.09	-0.05	-0.41	-0.21	1.00	0.77	0.84	0.31	0.08
# of tubules with many vacuoles	-0.17	-0.29	-0.09	-0.16	-0.09	-0.31	-0.21	0.77	1.00	0.47	-0.04	0.01
# of tubules with germ cell loss	0.08	-0.35	0.13	0.04	0.15	-0.58	-0.09	0.84	0.47	1.00	0.81	0.22
# of tubules with abnormal germ cells	0.19	-0.11	0.15	0.09	0.20	-0.27	0.08	0.31	-0.04	0.81	1.00	0.19
# of tubules with germ cell sloughing	0.28	0.01	0.23	-0.02	0.13	-0.12	0.08	0.08	0.01	0.22	0.19	1.00
10^6 sperm/mouse	0.28	0.53	0.51	0.32	0.44	0.21	0.54	-0.10	0.06	-0.11	-0.15	0.06
10^3 sperm/mg testis	0.07	0.17	0.27	0.11	0.24	0.05	0.27	0.09	0.30	-0.03	-0.17	0.06
10^3 sperm/seminiferous epithelium length	0.11	0.34	0.33	0.13	0.07	0.19	0.16	0.02	0.20	-0.10	-0.20	0.03
% normal morphology	-0.37	0.07	-0.11	-0.28	-0.10	0.19	-0.01	0.26	0.42	-0.12	-0.27	-0.09
% abnormal head shape	-0.16	-0.10	-0.20	0.03	-0.01	-0.04	-0.03	-0.26	-0.36	0.06	0.20	-0.25
% abnormal tail bending	0.60	0.05	0.38	0.42	0.21	-0.17	0.12	-0.09	-0.25	0.10	0.17	0.35
% broken tails	-0.24	-0.19	-0.35	-0.36	-0.27	-0.08	-0.30	-0.06	0.08	-0.01	0.00	-0.20
VCL t10	-0.47	-0.11	-0.36	-0.42	-0.28	0.04	-0.25	0.15	0.19	-0.23	-0.29	-0.23
% motile t10	0.13	0.34	0.16	0.31	0.22	0.06	0.25	-0.04	0.00	-0.26	-0.26	-0.28
% hyperactivated t10	-0.20	0.07	-0.09	-0.15	-0.14	0.24	-0.04	-0.15	-0.17	-0.29	-0.16	-0.15
% vigorous t10	-0.36	-0.11	-0.26	-0.29	-0.21	-0.04	-0.21	0.18	0.27	-0.18	-0.26	-0.21
VCL t90	-0.13	-0.07	-0.16	-0.29	-0.02	-0.19	-0.09	0.26	0.34	0.03	-0.08	0.02
% motile t90	0.33	0.45	0.31	0.41	0.30	0.13	0.35	0.10	0.13	-0.12	-0.12	0.00
% hyperactivated t90	-0.06	0.03	-0.02	-0.08	0.03	0.04	0.06	0.21	0.13	-0.08	-0.09	-0.01
% vigorous t90	-0.04	-0.09	-0.07	-0.20	0.05	-0.34	-0.07	0.28	0.37	0.08	-0.04	0.02
lactate	0.06	-0.26	-0.01	-0.16	0.19	-0.58	-0.01	0.15	0.27	0.01	-0.01	-0.01

	10^6 sperm/mouse	10^3 sperm/mg testis	10^3 sperm/seminiferous epithelium length	% normal morphology	% abnormal head shape	% abnormal tail bending	% broken tails	VCL t10	% motile t10	% hyperactivated t10	% vigorous t10	VCL t90	% motile t90	% hyperactivated t90	% vigorous t90	lactate
body weight	0.28	0.07	0.11	-0.37	-0.16	0.60	-0.24	-0.47	0.13	-0.20	-0.36	-0.13	0.33	-0.06	-0.04	0.06
mean testis weight	0.53	0.17	0.34	0.07	-0.10	0.05	-0.19	-0.11	0.34	0.07	-0.11	-0.07	0.45	0.03	-0.09	-0.26
mean epididymis + vas deferens weight	0.51	0.27	0.33	-0.11	-0.20	0.38	-0.35	-0.36	0.16	-0.09	-0.26	-0.16	0.31	-0.02	-0.07	-0.01
seminal vesicles weight	0.32	0.11	0.13	-0.28	0.03	0.42	-0.36	-0.42	0.31	-0.15	-0.29	-0.29	0.41	-0.08	-0.20	-0.16
# of seminiferous tubules/transverse section	0.44	0.24	0.07	-0.10	-0.01	0.21	-0.27	-0.28	0.22	-0.14	-0.21	-0.02	0.30	0.03	0.05	0.19
mean tubule radius	0.21	0.05	0.19	0.19	-0.04	-0.17	-0.08	0.04	0.06	0.24	-0.04	-0.19	0.13	0.04	-0.34	-0.58
seminiferous epithelium length/transverse section	0.54	0.27	0.16	-0.01	-0.03	0.12	-0.30	-0.25	0.25	-0.04	-0.21	-0.09	0.35	0.06	-0.07	-0.01
# of tubules with vacuoles	-0.10	0.09	0.02	0.26	-0.26	-0.09	-0.06	0.15	-0.04	-0.15	0.18	0.26	0.10	0.21	0.28	0.15
# of tubules with many vacuoles	0.06	0.30	0.20	0.42	-0.36	-0.25	0.08	0.19	0.00	-0.17	0.27	0.34	0.13	0.13	0.37	0.27
# of tubules with germ cell loss	-0.11	-0.03	-0.10	-0.12	0.06	0.10	-0.01	-0.23	-0.26	-0.29	-0.18	0.03	-0.12	-0.08	0.08	0.01
# of tubules with abnormal germ cells	-0.15	-0.17	-0.20	-0.27	0.20	0.17	0.00	-0.29	-0.26	-0.16	-0.26	-0.08	-0.12	-0.09	-0.04	-0.01
# of tubules with germ cell sloughing	0.06	0.06	0.03	-0.09	-0.25	0.35	-0.20	-0.23	-0.28	-0.15	-0.21	0.02	0.00	-0.01	0.02	-0.01
10^6 sperm/mouse	1.00	0.93	0.93	0.22	-0.24	-0.01	-0.22	-0.09	0.11	-0.10	-0.08	-0.11	0.24	-0.08	-0.09	0.02
10^3 sperm/mg testis	0.93	1.00	0.92	0.32	-0.29	-0.11	-0.16	0.00	0.04	-0.15	0.02	-0.05	0.15	-0.08	-0.02	0.19
10^3 sperm/seminiferous epithelium length	0.93	0.92	1.00	0.29	-0.28	-0.10	-0.09	0.06	0.12	-0.12	0.09	0.01	0.21	-0.08	0.02	0.07
% normal morphology	0.22	0.32	0.29	1.00	-0.59	-0.70	-0.10	0.45	0.21	0.11	0.37	0.41	0.33	0.44	0.27	-0.01
% abnormal head shape	-0.24	-0.29	-0.28	-0.59	1.00	-0.11	0.26	-0.35	-0.13	0.00	-0.32	-0.32	-0.26	-0.35	-0.28	0.11
% abnormal tail bending	-0.01	-0.11	-0.10	-0.70	-0.11	1.00	-0.39	-0.31	-0.16	-0.10	-0.25	-0.29	-0.16	-0.19	-0.17	-0.11
% broken tails	-0.22	-0.16	-0.09	-0.10	0.26	-0.39	1.00	0.13	0.01	-0.09	0.14	0.14	-0.10	-0.23	0.17	0.14
VCL t10	-0.09	0.00	0.06	0.45	-0.35	-0.31	0.13	1.00	0.26	0.39	0.91	0.74	0.21	0.50	0.74	0.12
% motile t10	0.11	0.04	0.12	0.21	-0.13	-0.16	0.01	0.26	1.00	-0.01	0.34	0.36	0.70	0.27	0.32	-0.03
% hyperactivated t10	-0.10	-0.15	-0.12	0.11	0.00	-0.10	-0.09	0.39	-0.01	1.00	0.23	0.06	-0.17	0.19	0.07	-0.12
% vigorous t10	-0.08	0.02	0.09	0.37	-0.32	-0.25	0.14	0.91	0.34	0.23	1.00	0.75	0.29	0.49	0.79	0.18
VCL t90	-0.11	-0.05	0.01	0.41	-0.32	-0.29	0.14	0.74	0.36	0.06	0.75	1.00	0.46	0.69	0.93	0.36
% motile t90	0.24	0.15	0.21	0.33	-0.26	-0.16	-0.10	0.21	0.70	-0.17	0.29	0.46	1.00	0.46	0.38	0.05
% hyperactivated t90	-0.08	-0.08	-0.08	0.44	-0.35	-0.19	-0.23	0.50	0.27	0.19	0.49	0.69	0.46	1.00	0.50	0.11
% vigorous t90	-0.09	-0.02	0.02	0.27	-0.28	-0.17	0.17	0.74	0.32	0.07	0.79	0.93	0.38	0.50	1.00	0.46
lactate	0.02	0.19	0.07	-0.01	0.11	-0.11	0.14	0.12	-0.03	-0.12	0.18	0.36	0.05	0.11	0.46	1.00
