# Supplementary material for: The Founder Strains of the Collaborative Cross Express a Complex Combination of Advantageous and Deleterious Traits for Male Reproduction
Source: G3 (Bethesda). 2015 Oct 13;5(12):2671–83. doi: 10.1534/g3.115.020172 (PMC4683640; doi:10.1534/g3.115.020172)
Supplement: Supporting Information [file supp_g3.115.020172_TableS5.zip › TableS5.pdf]

Table S5. P values for two-way ANOVA with age, strain and interaction between both.

trait	age	strain	age:strain
body weight	6.93E-14	1.74E-100	3.44E-09
mean testis weight	0.05920389	4.39E-50	0.3621966
mean epididymis + vas deferens weight	0.01243687	4.73E-36	0.5751398
seminal vesicles weight	4.24E-11	1.36E-39	4.74E-07
# of seminiferous tubules/transverse section	0.9551763	1.03E-26	0.0209278
mean tubule radius	0.001712782	1.52E-39	0.06757473
seminiferous epithelium length/transverse section	0.300091	1.35E-25	0.05566402
# of tubules with vacuoles	1.34E-06	1.72E-21	0.02144394
# of tubules with many vacuoles	0.005713671	7.36E-24	0.004638549
# of tubules with germ cell loss	0.03587775	0.00133563	0.2213439
# of tubules with abormal germ cells	0.4439329	0.001249672	0.8450332
# of tubules with germ cell sloughing	0.4595321	0.003646249	0.000462052
10^6 sperm/mouse	0.1658613	7.35E-14	0.3935378
10^3 sperm/mg testis	0.4894727	2.30E-09	0.4589633
10^3 sperm/seminiferous epithelium length	0.3679104	1.66E-05	0.4289896
% normal morphology	0.652714	1.50E-11	0.4312549
% abnormal head shape	0.000342395	4.58E-09	0.3884816
% abnormal tail bending	7.32E-07	2.57E-12	0.007488992
% broken tails	0.000186813	0.1802647	0.8745298
% motile t10	0.1085157	1.18E-08	0.003231376
VCL t10	0.3042305	1.62E-23	0.08054154
% vigorous t10	0.7610701	9.64E-16	0.03961111
% hyperactivated t10	0.3126049	1.10E-05	0.6120385
% motile t90	0.4138724	4.72E-15	0.1714224
VCL t90	0.03083604	1.68E-24	0.03145291
% vigorous t90	0.05365229	3.61E-25	0.117164
% hyperactivated t90	0.008905577	1.36E-09	0.000717168
lactate	0.06234563	0.000113436	0.02300906
